# Strengthening Early Childhood Development Programs and Interventions in Low‐ and Middle‐Income Countries: Systematic Review of Interventions

**DOI:** 10.1111/mcn.70244

**Published:** 2026-09-01

**Authors:** Sonia Hernández‐Cordero, Bianca Franco‐Lares, Mauricio Hernández‐F, Marlene Peters‐Castilla, Vania Lara‐Mejía, Pilar Echandi‐Ruíz, Rafael Pérez‐Escamilla

**Affiliations:** ^1^ Research Institute for Equitable Development (EQUIDE) Universidad Iberoamericana Mexico City Mexico; ^2^ Francisco Xavier Clavigero Library Universidad Iberoamericana Mexico City Mexico; ^3^ Department of Social and Behavioral Sciences Yale School of Public Health New Haven Connecticut USA

**Keywords:** early childhood development, implementation science, interventions, low‐ and middle‐income countries, nurturing care

## Abstract

Early childhood development (ECD) is essential across life courses, yet many children lack adequate nurturing care needed for optimal ECD. The Nurturing Care Framework (NCF) is designed to optimize ECD outcomes through evidence‐based programs and interventions. Therefore, the NCF is key to identify and characterize programs and interventions and to determine key challenges and enabling factors for their effective large‐scale implementation. A systematic review following the PRISMA reporting guidance was conducted. PubMed Central, Web of Science, Science Direct, Scopus and MEDLINE databases were used. Studies in English and Spanish, from 2014–2024, 2014 to 2024, addressing interventions or programs based on NFC in low‐ and middle‐income countries (LMICs)(LMICs) were included. Exclusion criteria were studies focused only on caregivers. Quality was assessed with the NICE checklist. A narrative synthesis was conducted by NCF domains. A total of 34 studies were included (from 784 identified), representing five ECD programs and 29 interventions across 19 LMICs. Overall, most elements delivered were aligned with the early learning and nutrition NCF domains. Key implementation challenges and enablers identified were related to logistical constraints, heterogeneity in program fidelity, resistance among implementers to adopt new practices, and low attendance to sessions. Based on 10 interventions, the average annual cost per child was $197.4; 6 reported that the interventions were cost‐effective. Despite the need for comprehensive ECD interventions and programs, these remain fragmented and insufficiently address the NCF domains. Addressing these evidence implementation gaps and assessing their cost‐effectiveness is essential to inform policy and guide decision‐making for scalable and sustainable ECD programs in LMICs.

## Key Messages

- The majority of the included early childhood development (ECD) interventions and programs, addressing the Nurturing Care Framework of opportunities for early learning and adequate nutrition domains, showed a positive impact on outcomes, supporting their continuation and scale‐up in practice.
- The ECD interventions identified across studies and contexts in low‐ and‐middle income countries are heterogeneous, limiting comparability.
- Research in integrated ECD interventions and programs addressing multiple components and integrated Nurturing Care Framework domains is needed to achieve meaningful impact.
- Important gaps persist in ECD program implementation reporting, specially regarding population reach, adoption across settings, and long‐term sustainability, which hinders informed decision‐making by program managers and policy makers.
- Evidence on cost‐effectiveness of diverse ECD interventions and programs across settings is scarce and methodologically diverse, constraining the opportunity for decision‐makers to prioritize investments in integrated ECD programs and highlighting the need for more robust and comparable economic evidence to guide resource allocation.

## 1 Introduction

The period from conception through the first 5 years of life is foundational for early childhood development (ECD) (Black et al. 2017; Britto et al. 2017; Draper et al. 2024; Nores et al. 2024; Richter et al. 2017). These foundational skills are crucial for long‐term human development as they shape the brain pathways for cognition, self‐esteem, emotional regulation, impulse control, decision‐making, soft skills, and planning abilities (Black et al. 2017; Britto et al. 2017; Draper et al. 2024; Nores et al. 2024; Richter et al. 2017). During this period, the capacity for humans to grow healthy also gets established (Black et al. 2017; Britto et al. 2017; Draper et al. 2024; Nores et al. 2024; Richter et al. 2017).

ECD is an essential part of the intergenerational human development process. Investing in ECD helps break poverty cycles, reduce inequities (Heckman 2011; Serván‐Mori et al. 2022), and contributes to national development (Nguyen et al. 2018). Furthermore, ECD investments contribute to improving well‐being and equity in the most disadvantaged and vulnerable population groups (Baek et al. 2023; Nguyen et al. 2018; Serván‐Mori et al. 2022), and are highly cost‐effective (Heckman 2011) at the same time that they advance human rights.

In low‐ and middle‐income countries (LMICs), it is estimated that children with poor ECD have, on average, 19.8% lower annual incomes in adulthood (Isquith‐Dicker et al. 2021; Richter et al. 2017). Around 43% of children under 5 years of age in LMICs, which translates into 250 million children, do not achieve their developmental potential because of adverse circumstances such as poverty, malnutrition, and lack of care and stimulation (Isquith‐Dicker et al. 2021). This long‐standing problem particularly affects the 1.2 billion children worldwide who experience multidimensional poverty (Save the Children 2023).

ECD investments should be guided by the Global Nurturing Care Framework for Early Childhood Development (NCF) (Buccini et al. 2021; WHO, UNICEF, & World Bank Group 2018). The NCF, resulting from the 2016 Lancet Early Childhood Development Series, comprises five main domains: health, nutrition, safety and security, responsive care, and early learning (Black et al. 2017; Britto et al. 2017; WHO, UNICEF, & World Bank Group 2018). Social and other environmental contexts need to be considered when implementing ECD programs (Likhar et al. 2022). These contextual factors influence ECD through different pathways, including expected gender roles, socio‐economic circumstances, family structures, intimate partner violence, opportunities for early stimulation and learning, access to outdoor areas, and neighborhood safety (Abboah‐Offei et al. 2022; Atashbahar et al. 2022; Black et al. 2017; Britto et al. 2017; Likhar et al. 2022); and in turn, shape the implementation and scalability of ECD interventions.

Therefore, it is essential to identify, characterize, and determine key challenges and enabling factors for implementing effective large‐scale ECD programs or interventions in LMICs. However, existing evidence remains fragmented, with limited integration of implementation processes, delivery strategies, and cost‐effectiveness within a unified analytical framework such as the NCF. This review adds to previous work in this area (Bernard van Leer Foundation 2011; Buccini et al. 2021; Cavallera et al. 2019; Coore‐Hall et al. 2023; Khan et al. 2018; Milman et al. 2018; Pérez‐Escamilla et al. 2018; Rao and Kaul 2018; Richter et al. 2017; Richter and Samuels 2018; Torres et al. 2018) by systematically identifying and characterizing ECD programs and interventions in LMICs based on the NCF, identifying implementation challenges and facilitators and summarizing programs' cost‐effectiveness data. The primary objective of this systematic review was to identify, characterize, and synthesize programs or interventions implemented in LMIC to improve ECD, addressing all or some components of the NCF. The secondary objective was to analyze the cost‐effectiveness and to identify key implementation challenges and enabling factors for delivering the identified programs and interventions. We expect that this review will help inform the decision‐making process related to ECD investments.

## 2 Methods

The protocol for this systematic review was registered on PROSPERO (CRD42024554760).

### 2.1 Search Strategy

This systematic review was guided by the Preferred Reporting Items for Systematic Reviews and Meta‐analyses (PRISMA) framework (Page et al. 2021). Five electronic databases were used for the information search (Web of Science, Scopus, PubMed Central, ScienceDirect, and MEDLINE), with a time frame of 10 years (2014–2024). The search strategy was based on search terms and keywords used in previous systematic reviews (Buccini et al. 2023; Hurt et al. 2018; Jeong et al. 2021; Zhang et al. 2021), combined with MeSH (Medical Subject Headings) terms, and was conducted in English and Spanish. Relevant literature was identified following three search algorithms summarized in Supporting Information 1. Reference lists of relevant studies and reviews were examined to identify additional studies that may have been missed. The searches were conducted by one of the co‐authors (BF‐L).

### 2.2 Eligibility Criteria

Table 1 presents the eligibility criteria and study characteristics considered in this systematic review, comprising both inclusion and exclusion study selection criteria. Briefly, studies were eligible for inclusion if they evaluated ECD programs, policies, or interventions targeting the period from pregnancy through children under 5 years of age in LMICs. Randomized controlled trials, longitudinal studies, and quasi‐experimental studies were included. Studies excluded were systematic reviews, meta‐analyses, and study protocols. Studies that did not specifically address ECD outcomes or that exclusively targeted mothers without reporting follow‐up data on child outcomes were also excluded, as well as studies focusing on specific clinical subgroups (e.g., preterm infants).

**Table 1:** Eligibility criteria and characteristics for systematic reviews on early childhood development programs and interventions.

Eligibility criteria	
Inclusion criteria	
Study design	Observational, longitudinal, quasi‐experimental, randomized controlled trial, impact evaluation, cost‐effectiveness evaluation, and qualitative peer‐reviewed studies.
Type of intervention	– Programs, policies, and interventions promoting early childhood development, targeting the period of pregnancy through children under five years of age.
	– Early childhood development interventions: interventions applied to children or mother‐child dyads to promote or improve skills such as language, cognition, social, emotional, and motor development, and psychosocial well‐being. Interventions should include all or some domains of the Nurturing Care Framework (e.g., health, nutrition, safety and security, responsive care, or opportunities for early learning).
Target population	Mother and children (under five years old)
Context	Studies addressing programs, interventions, or policies to promote early childhood development in low and middle‐income countries.
Language	English and Spanish.
Time frame	2014–2024
Exclusion criteria	
Study design	Systematic review, metaanalysis, study protocols, trial registrations and abstracts.
Type of intervention	Studies that did not specifically address promoting ECD.
Target population	Studies exclusively targeted mothers, parents, caregivers without reporting follow‐up data on child development and health outcomes and those focusing on specific clinical subgroups, such as preterm infants
Outcome	Studies that not report child development and health outcomes

We defined a program as a structured, long‐term strategy with a broad reach funded with public resources, whereas an intervention was defined as a specific, time‐bound action implemented in a defined context (Rossi et al. 2004).

### 2.3 Outcomes

Based on the NCF domains, this review included outcome indicators for children under 5 years old. These outcomes were included regardless of the assessment tools or instruments utilized for their measurement. The outcomes were categorized as: cognitive development (e.g., cognitive development, literacy, numeracy, reading, and cognitive scores); language (e.g., communication skills); motor development (e.g., fine and gross motor skills); socio‐emotional (e.g., social interactions, responsive caregiving). Malnutrition indicators (e.g., stunting and/or wasting) were included as part of the adequate nutrition domain.

When reported, implementation challenges and enabling factors for delivering the identified programs and interventions were included as a secondary outcome.

### 2.4 Study Selection

Following the de‐duplication process, titles and abstracts were independently screened by two co‐authors (BF‐L and VL‐M). Subsequently, full‐text articles were assessed for eligibility based on the inclusion and exclusion criteria (Table 1). Discrepancies were identified and resolved through discussion; when consensus was not reached, a third co‐author (SH‐C) facilitated the consensus.

### 2.5 Data Extraction

Data were extracted using a previously tested Excel spreadsheet. Data extraction was conducted independently by three co‐authors (BF‐L, VL‐M, SH‐C), following a pilot test on five randomly selected studies to refine the process. The extraction form included: authors, country, study design, study population, implementation context (e.g., delivery method, exposure frequency, exposure length), NCF domains addressed (e.g., good health, adequate nutrition, responsive caregiving, opportunities for early learning, security and safety), effectiveness outcomes (e.g., odds ratio (OR), β coefficients, or mean differences (MD), with 95% confidence intervals (adjusted or crude)), cost and/or cost‐effectiveness data when available, and barriers and facilitators to implementation.

### 2.6 Quality Assessment

The retrieved articles were evaluated using the National Institute for Health and Care Excellence (NICE) checklists for quantitative or qualitative studies (National Institute for Health and Care Excellence 2012). The NICE quality standards were selected because they provide clear structured criteria to assess internal and external validity.

At least two of four reviewers (BF‐L, VL‐M, SH‐C, MP‐C) independently assessed each study following prior standardization. Discrepancies were resolved through consensus. Studies were classified as having adequate, moderate, or low quality based on their internal and external validity.

### 2.7 Data Analysis

Extracted data were synthesized according to child development and nutrition outcomes and grouped following the NCF domains. A narrative synthesis approach was used, focusing on intervention and program effects. No quantitative synthesis of effect sizes was conducted due to substantial heterogeneity in outcomes and measurement instruments across studies. Instead, effect estimates (e.g., OR, β coefficients, and MD) were extracted and reported as presented in the original articles. Additionally, intervention and program characteristics (e.g., delivery modality, frequency, and duration) were comprehensively synthesized.

In addition, the RE‐AIM (Reach, Effectiveness, Adoption, Implementation, and Maintenance) framework (Jilcott et al. 2007) was used as an analytical approach to examine implementation and scalability of the included programs and interventions. This analysis was restricted to studies reporting both implementation‐related information and cost data. Data were extracted and synthesized across RE‐AIM dimensions, including population reach, intervention effectiveness, adoption across settings, implementation processes (e.g., fidelity, adaptations, and delivery challenges), and maintenance and scalability. Consistent with this framework, key implementation challenges and enabling factors were identified and interpreted.

### 2.8 Cost Analysis and Synthesis of Cost‐Effectiveness Evidence

We extracted total intervention costs and sample sizes from studies reporting cost‐effectiveness results and calculated cost per participant when not directly reported. For comparison purposes, all cost estimates were converted to constant 2023 US dollars to enhance comparability.

Given that interventions were implemented in different time periods, direct comparison of reported costs in nominal US dollars would be misleading due to inflation and changes in currency value over time. To address this, when costs were reported in local currencies, values were adjusted using country‐specific inflation indices and then converted to 2023 US dollars using purchasing power parity (PPP)‐adjusted exchange rates. When costs were originally reported in US dollars, they were adjusted for inflation and converted to 2023 US dollars following the same approach. When the year of cost estimation was not specified, we assumed it corresponded to the year of publication.

Given the heterogeneity in intervention designs, outcomes, and analytic perspectives, a standardized cost‐effectiveness metric was not used, and results were not pooled across studies. Instead, we synthesized the available evidence as reported by the original studies. We recorded whether the studies conducted a cost‐effectiveness analysis and what the result of that analysis was (e.g., whether the intervention was cost‐effective or not), and extracted incremental cost‐effectiveness ratios (ICERs) (Gold et al. 1996) when available. Effectiveness was defined based on reported impacts on child development outcomes. Due to variation in outcome measures and evaluation frameworks, cost‐effectiveness results are presented descriptively and should not be interpreted as directly comparable across studies. For example, the intervention reported by Attanasio et al. (2022) addresses four domains of the NCF, whereas the one reported by Spier et al. (2020) addresses only a single domain. Therefore, it is to be expected that the former intervention would incur higher costs than the latter.

## 3 Results

The included studies comprised both ECD interventions and programs as defined in the methods section.

### 3.1 Study Characteristics

**Figure 1:**
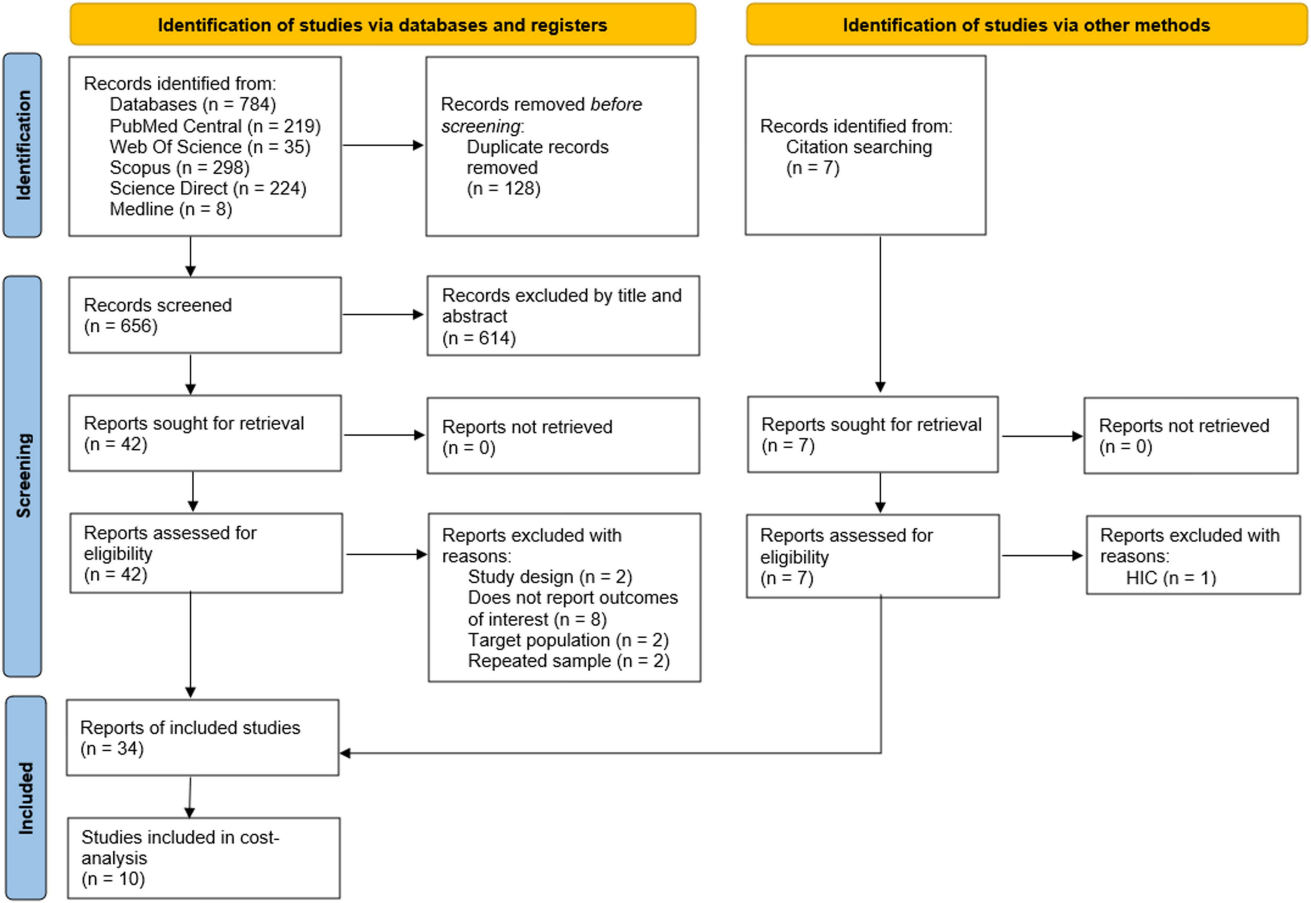
Preferred reporting items for systematic reviews and meta‐analyses (PRISMA) flow diagram of systematic review on early childhood development programs and interventions.

Initially, 784 articles were identified. After removing 128 duplicates, a total of 656 articles were screened by title and abstract based on inclusion and exclusion criteria, resulting in 42 articles selected for full‐text assessment. Additionally, seven articles were identified through backward citation chaining and included in the full‐text review. After full‐text assessment, 15 articles did not meet the eligibility criteria and were excluded. The reasons for exclusion are described in Supporting Information 2. A total of 34 articles were included in this systematic review, of which 10 included cost‐effectiveness data (Figure 1).

Regarding study design, the majority of included studies were randomized trials. Sixteen were cluster randomized controlled trials (CRCTs) (Ahmed et al. 2023; Attanasio et al. 2022; Baek et al. 2023; Bliznashka et al. 2022; Desmond et al. 2023; Gaidhane et al. 2023; Grantham‐McGregor et al. 2020; Hossain et al. 2022, 2024; Jensen et al. 2021; Luo et al. 2019; Nuño et al. 2022; Shi et al. 2020; Sylvia et al. 2022; Tofail et al. 2018; Weisleder et al. 2018), and six were randomized controlled trials (RCTs) (Bemanalizadeh et al. 2022; López García et al. 2021; Santos et al. 2022; Skeen et al. 2023; Spier et al. 2020; Waechter et al. 2022). Additionally, 11 were non‐randomized or observational studies, including quasi‐experimental (*n* = 6) (González‐Fernández et al. 2020; Leighton et al. 2023; M. Li et al. 2024; Rosales et al. 2019; Saori et al. 2019; Zhou et al. 2019), cohort (*n* = 2) (Koshy et al. 2024; Viegas Da Silva et al. 2022), cross‐sectional (*n* = 2) (Kamiya and Nomura 2023; Russell et al. 2022), pre‐post (Pisani et al. 2016), and qualitative case (Buccini et al. 2021) studies. Supporting Information 3 summarizes the main characteristics of the included studies.

**Figure 2:**
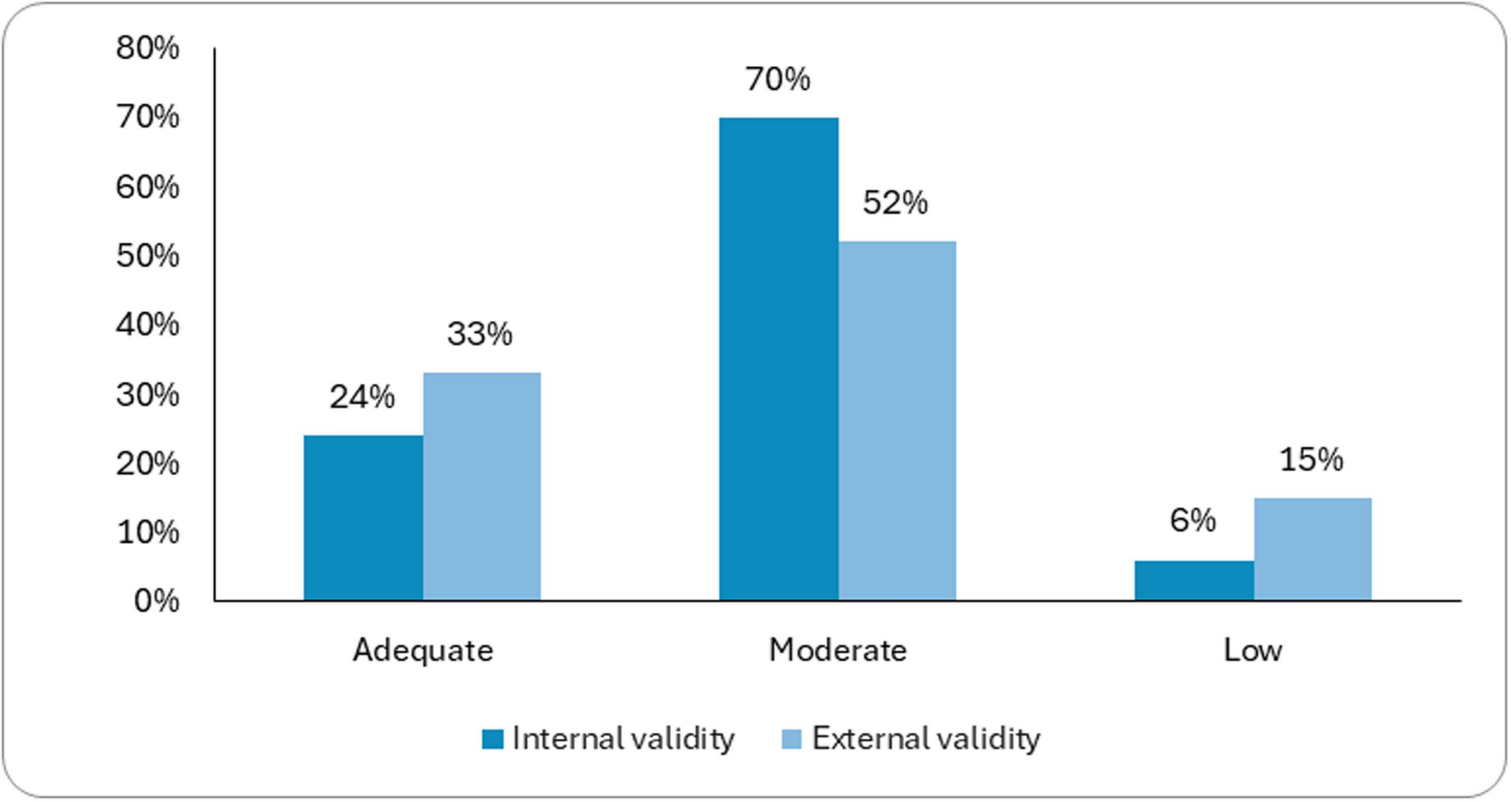
Quality assessment of included quantitative studies (*n* = 33) according to NICE guidelines. Systematic review on early childhood development programs and interventions.

In the quality assessment, for internal validity, of the 33 quantitative studies included, 24% were assessed as adequate, 70% as moderate, and 6% as low quality. Regarding external validity, 33%, 52%, and 15% were assessed as adequate, moderate, and low, respectively. The only qualitative study included was assessed as having adequate quality (Figure 2). Overall, most studies were of moderate methodological quality, with common limitations related to reporting and study design. The main internal validity limitations in the included studies were that the allocation to groups was not well described (15%), there was insufficient or unclear description of the intervention or control groups (33%), and failure to report the risk of contamination between the study groups (48.5%). External validity limitations primarily included limited detail about the study populations (44%).

### 3.2 Where Did the Evidence Come From?

**Figure 3:**
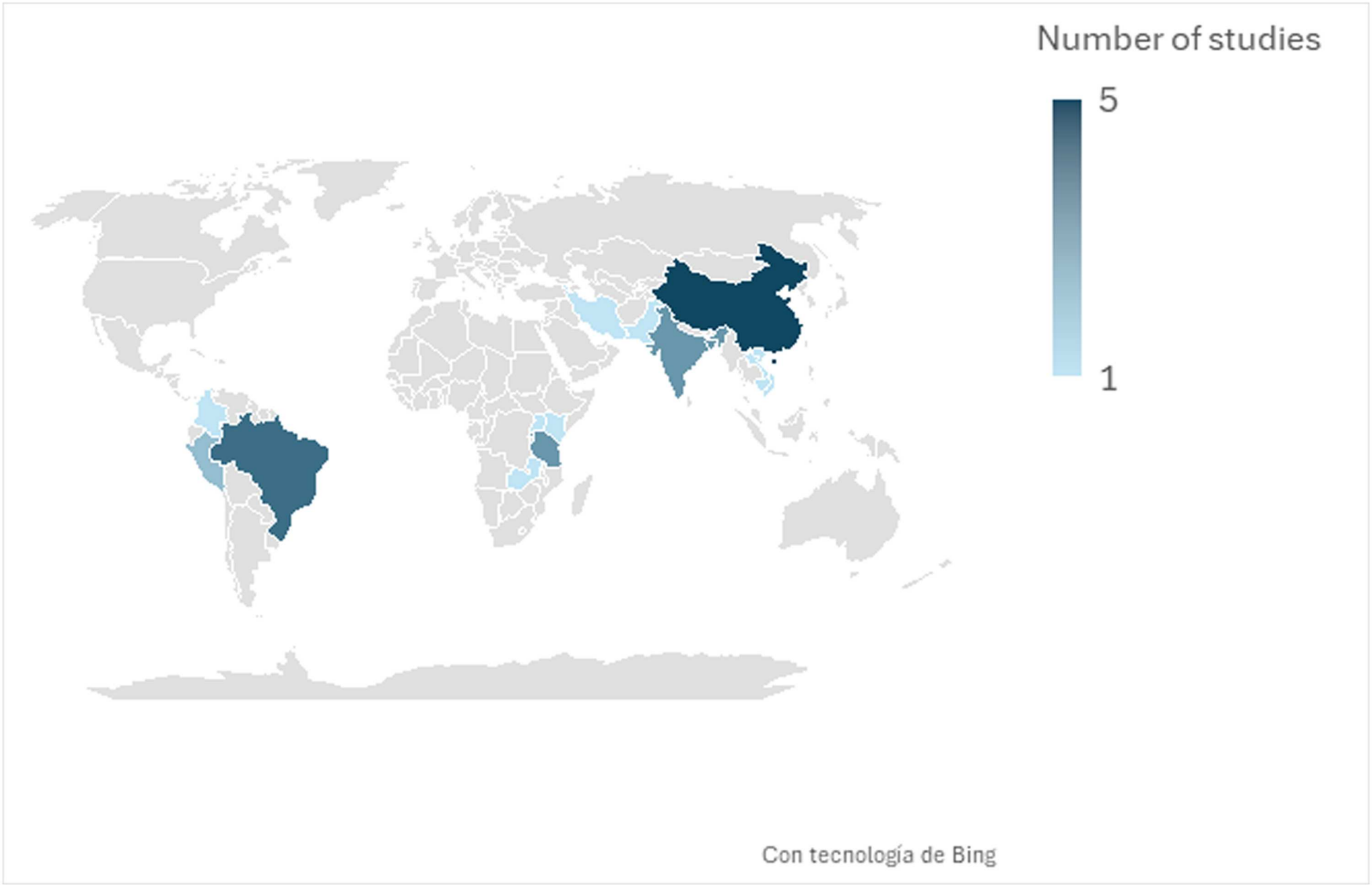
Source countries of identified interventions and programs on early childhood development (2014–2024). Systematic review on early childhood development programs and interventions.

The systematic review included 34 studies, five of which corresponded to ECD programs (Buccini et al. 2021; Kamiya and Nomura 2023; Russell et al. 2022; Santos et al. 2022; Viegas Da Silva et al. 2022) and 29 to ECD interventions, conducted in 19 LMICs. The studies represented different world regions including the Region of the Americas (AMR) (*n* = 5); Africa Region (AFR) (*n* = 5); Western Pacific Region (WPR) (*n* = 3); Eastern Mediterranean Region (EMR) (*n* = 2); South‐East Asian Region (SEAR) (*n* = 3); and European Region (EUR) (*n* = 1) (Figure 3). Overall, the evidence was geographically diverse, although unevenly distributed across regions.

### 3.3 Domains of the Nurturing Care Framework: What Is Being Delivered?

Table 2 synthesizes the elements of the interventions and programs included, categorized according to NCF domains. Most of the NCF elements delivered were aligned with the child health, opportunities for early learning, and adequate nutrition domains.

**Table 2:** Elements of the interventions or programs being delivered by the nurturing care framework domain. Systematic review on early childhood development programs and interventions.

Elements being delivered by the program or intervention by domain				
Good health	Adequate nutrition	Responsive caregiving	Opportunities for early learning	Security and safety
Child age‐appropriate ECD counseling on health for families (Buccini et al. 2021; González‐Fernández et al. 2020; Rosales et al. 2019).	Child age‐appropriate ECD counseling on nutrition for families (Attanasio et al. 2022; Bliznashka et al. 2022; Rosales et al. 2019; Zhou et al. 2019).	Child age‐appropriate ECD counseling on responsive care for families (Weisleder et al. 2018; Zhou et al. 2019).	Early stimulation interventions through group sessions of caregiver‐child play activities (Bliznashka et al. 2022; Zhou et al. 2019).	Child age‐appropriate ECD counseling on child protection (Buccini et al. 2021; Jensen et al. 2021).
Information on infant health (González‐Fernández et al. 2020; Russell et al. 2022; Zhou et al. 2019).	Nutrition workshops focused on awareness of child needs “conscious nutrition” (González‐Fernández et al. 2020).	Promotion and collaboration in the exercise of parenting focuses on strengthening families' roles in the care of children and affective and community bonds (Pisani et al. 2016).	Child development toys and books (M. Li et al. 2024; Rosales et al. 2019).	Promotion and collaboration in the exercise of families in child protection (Buccini et al. 2021; Leighton et al. 2023).
Information on maternal mental health (Baek et al. 2023; Skeen et al. 2023).	Mothers' education on increasing dietary diversity (Grantham‐McGregor et al. 2020).	Information about how to improve the nurturing care environment in which children thrive (Bliznashka et al. 2022; Grantham‐McGregor et al. 2020).	Promotion and collaboration in strengthening families' roles in children's education (Bemanalizadeh et al. 2022; Leighton et al. 2023; Shi et al. 2020; Sylvia et al. 2022; Waechter et al. 2022).	Information on gender‐based violence and empowerment (Desmond et al. 2023; Jensen et al. 2021).
Promotion of sanitation (Grantham‐McGregor et al. 2020; Nuño et al. 2022).	Nutritional supplement (Attanasio et al. 2022; Bliznashka et al. 2022; González‐Fernández et al. 2020).	Information on parenting capabilities (Attanasio et al. 2022; Rosales et al. 2019; Shi et al. 2020).	Information about developmentally appropriate play and talk activities with their child, including providing educational toys and books (Attanasio et al. 2022; Bliznashka et al. 2022; Rosales et al. 2019; Sylvia et al. 2022).	Practical demonstrations in group sessions to educate and empower the mothers (Ahmed et al. 2023; Baek et al. 2023).
Hygiene education component conveyed three main messages: (i) keeping kitchen environments clean; (ii) washing mother and child's hands with soap at key moments (e.g., before preparing meals); and (iii) household water treatment (Nuño et al. 2022).	Information on basic nutrition (Luo et al. 2019; Tofail et al. 2018; Zhou et al. 2019).	Promotion of stimulation and nurturing care (Bliznashka et al. 2022; Russell et al. 2022; Shi et al. 2020).	Information about reading to children (Pisani et al. 2016; Weisleder et al. 2018).	
10 L vessel with a lid and tap and a regular supply of sodium dichloroisocyanurate tablets for water disinfection (Tofail et al. 2018).	Information on maternal nutrition (Buccini et al. 2021).		Promotion of early learning (Leighton et al. 2023).	
Construction and maintenance of latrines and potties for children younger than 3 years and a sani‐scoop for removing feces from the compound (Tofail et al. 2018).	Lipid‐based nutrient supplements for children aged 6–24 months, along with counseling on breastfeeding and complementary feeding practices (Pisani et al. 2016; Tofail et al. 2018).		Teaching caregivers how to engage in age‐appropriate activities with their children, such as reading, storytelling, and singing, to improve cognitive, motor, and socioemotional skills (Bemanalizadeh et al. 2022; Leighton et al. 2023; Sylvia et al. 2022; Waechter et al. 2022).	
Two hand‐washing stations, one with a 40 L water reservoir placed near the latrine and a 16 L reservoir for the kitchen (Tofail et al. 2018).	Information about age‐appropriate feeding practices, dietary diversity, meal frequency, and nutritional supplementation (Grantham‐McGregor et al. 2020; Luo et al. 2019).		Book‐lending library and monthly parent workshops with a facilitator (1 h) who guided discussion about reading aloud and other opportunities for interacting with children, such as playing and talking during everyday routines (Weisleder et al. 2018).	
Information about immunization, hygiene, and healthy eating and sleeping behavior (Luo et al. 2019).	General and nutritional messages (Hossain et al. 2022).		Tool kit to develop skills in the preschool years through play and joyful learning at home (Saori et al. 2019).	
Monthly visits during follow‐up to reinforce hygiene education and the correct maintenance of improved biomass cookstoves (Nuño et al. 2022).			Providing preschool teachers with specific structured language and learning inputs from early childhood curriculum (Saori et al. 2019).	
			Positive parenting and early stimulation techniques such as reading aloud and talking during everyday routines (Weisleder et al. 2018).	

Abbreviation: ECD: early childhood development.

^†^ Elaborated by the authors based on the available information of the included studies.

Elements delivered among child health included information on infant health, maternal mental health, hygiene education, and promotion of sanitation. Adequate nutrition included information on nutrition for families, counseling on breastfeeding and complementary feeding practices, and provision of nutritional supplements. Regarding opportunities for early learning elements included caregiver‐child activities, by providing educational toys, books, guidance about reading aloud to children, and the provision of preschool teachers with structured language and learning inputs. The safety and security domain was the least addressed, when provided it included information on gender‐based violence, women's empowerment, and child protection. Both interventions and programs were typically delivered through direct contact with mothers or caregivers, workshops, or group sessions.

### 3.4 How Were Interventions and Programs Delivered?

Different strategies for delivering interventions and programs were identified: home visiting, face‐to‐face group sessions at a community center or a village clinic, combined modalities (e.g., home visiting and face‐to‐face group sessions), digital messaging via WhatsApp, and preschool‐based interventions. Table 3 summarizes the delivery strategies of the studies included.

**Table 3:** Delivery strategies for the components of the early child development programs and interventions. Systematic review on early childhood development programs and interventions.

	Delivery method				
	Home visiting	Face‐to‐face group sessions at community centers or village clinics	Mixed	Preschools	Via whatsApp
Frequency	Bi‐weekly (Luo et al. 2019), weekly (Buccini et al. 2021; González‐Fernández et al. 2020; Jensen et al. 2021; Viegas Da Silva et al. 2022), every 2 weeks (Hossain et al. 2022), monthly (Bliznashka et al. 2022; Waechter et al. 2022), every 2 months (M. Li et al. 2024), weekly visits for the first 6 months and every 2 weeks for the next 18 months (Tofail et al. 2018).	3 to 4 days a week (Zhou et al. 2019); weekly (Ahmed et al. 2023; Leighton et al. 2023); during routine well‐child visits at 2 and 6 months of age (Shi et al. 2020); monthly (Weisleder et al. 2018); third trimester of pregnancy, one group session at 2‐6 weeks old and one every three months for the first year of age (Bemanalizadeh et al. 2022); every 3 months (Rosales et al. 2019).	Weekly home and group sessions (Grantham‐McGregor et al. 2020); every 2 weeks home and group sessions; every 2 weeks home visiting and monthly group sessions (Gaidhane et al. 2023); every 2 months home visiting and every month group sessions (López García et al. 2021); monthly home visits and weekly group sessions (Attanasio et al. 2022); 8 group sessions during pregnancy, one home visit after childbirth, 11 home visits during 1 year (Baek et al. 2023)	Daily (Saori et al. 2019; Spier et al. 2020).	Weekly sessions (Skeen et al. 2023).
Length of the session	40 to 60 min (Hossain et al. 2022); 45 to 60 min (Viegas Da Silva et al. 2022); 60 min (Jensen et al. 2021); 2 h.	45 min (Bemanalizadeh et al. 2022); 1 h (Ahmed et al. 2023).	Group session 90 min and home visit 1 h (Attanasio et al. 2022; Grantham‐McGregor et al. 2020; López García et al. 2021).		1‐2 h (Skeen et al. 2023).
Length of the program or intervention	3 months (Jensen et al. 2021); 6 months; 1 year (Hossain et al. 2022; Kamiya and Nomura 2023; Luo et al. 2019; Waechter et al. 2022); 2 years (Bliznashka et al. 2022; M. Li et al. 2024; Tofail et al. 2018); 3 years; from uthero to 5 years of child's age (Russell et al. 2022); from pregnancy until 3 years of child's age (Buccini et al. 2021).	6 months (Ahmed et al. 2023); 1 year (Shi et al. 2020); 14 months (Rosales et al. 2019); from uthero to 1 year of child age (Bemanalizadeh et al. 2022); 2 years (Zhou et al. 2019).	8 months (López García et al. 2021); 18 months (Baek et al. 2023); 1 year (Sylvia et al. 2022); 2 years (Gaidhane et al. 2023; Grantham‐McGregor et al. 2020).	2 years (Saori et al. 2019; Spier et al. 2020).	6 weeks (Skeen et al. 2023).
Who delivers the program or intervention	Local health worker (Bliznashka et al. 2022; Hossain et al. 2022; Luo et al. 2019); trained community‐based non‐specialists (Jensen et al. 2021; Waechter et al. 2022); country doctor who received a series of training curricula (M. Li et al. 2024).	Community health workers (Leighton et al. 2023), child development experts (Shi et al. 2020).	Community health workers (Gaidhane et al. 2023), local women with high school degree (Attanasio et al. 2022), child development experts (Sylvia et al. 2022).	Child development experts (Spier et al. 2020).	Intervention facilitators (Skeen et al. 2023).
To whom is the program or intervention delivered	Caregivers and their child (Bliznashka et al. 2022; Hossain et al. 2022; Jensen et al. 2021; Luo et al. 2019; Tofail et al. 2018; Waechter et al. 2022); pregnant women (Baek et al. 2023; Bemanalizadeh et al. 2022; Buccini et al. 2021); 3–4 years old children (Kamiya and Nomura 2023).	Caregivers and their child (Bemanalizadeh et al. 2022; Rosales et al. 2019; Shi et al. 2020; Weisleder et al. 2018), parents of children under 5 years old (Leighton et al. 2023).	Caregivers and their child (Gaidhane et al. 2023; López García et al. 2021).	Preschoolers 4 years old (Spier et al. 2020), preschool teachers and parents (Saori et al. 2019).	Caregivers (Skeen et al. 2023).

^†^ Elaborated by the authors based on the available information of the included studies.

Regarding frequency, home visiting interventions ranged from weekly (Brazil, Rwanda, Peru) (Desmond et al. 2023; González‐Fernández et al. 2020; Viegas Da Silva et al. 2022), bi‐weekly (China) (Zhou et al. 2019), monthly (Perú, Bangladesh, Pakistan) (Bliznashka et al. 2022; Hossain et al. 2024; Nuño et al. 2022) or bimonthly (China) (M. Li et al. 2024). Similarly, delivery frequency for interventions combining home visiting with face‐to‐face group sessions ranged from weekly to monthly (India, Kenya, Colombia, Vietnam) (Attanasio et al. 2022; Baek et al. 2023; Gaidhane et al. 2023; Grantham‐McGregor et al. 2020; López García et al. 2021). In contrast, interventions in facility‐based preschools were delivered daily (Rwanda) (Saori et al. 2019; Spier et al. 2020).

The personnel responsible for delivering the interventions and programs consisted mainly of health professionals with or without ECD specialists. Home visits were made by local health workers (Bangladesh) (Hossain et al. 2022), trained community‐based non‐specialists (Rwanda, Colombia) (Attanasio et al. 2022; Desmond et al. 2023), or trained country doctors (China) (M. Li et al. 2024); while face‐to‐face group sessions were led by community health workers and child development experts (Tanzania, Colombia, China) (Attanasio et al. 2022; Leighton et al. 2023; Shi et al. 2020; Sylvia et al. 2022). The length of the sessions varied according to the delivery method. Home visiting lasted on average 40–60 min (Rwanda, Bangladesh, Pakistan, India, China) (Bliznashka et al. 2022; Desmond et al. 2023; Gaidhane et al. 2023; Grantham‐McGregor et al. 2020; Hossain et al. 2022; Spier et al. 2020; Zhou et al. 2019). Similarly, face‐to‐face group sessions were on average 40–60 min long (Iran, Uganda) (Ahmed et al. 2023; Bemanalizadeh et al. 2022), while combined delivery methods lasted 60–90 min (Colombia, India, Kenya) (Attanasio et al. 2022; Grantham‐McGregor et al. 2020; López García et al. 2021). Regarding length, home visiting interventions range from 3 months to 3 years, face‐to‐face group sessions, and combined interventions from 6 months to 2 years; while the length of home visiting programs ranging from pregnancy and their child up to three (Brazil) (Buccini et al. 2021; Santos et al. 2022) or 5 years old (Tanzania) (Russell et al. 2022).

In addition, the delivery settings for interventions involving face‐to‐face group sessions were diverse ranging from ECD centers at village level (China) (Zhou et al. 2019), community centers (Vietnam) (Baek et al. 2023) and primary healthcare clinics at the township/village levels (China, Tanzania, Colombia) (Attanasio et al. 2022; Russell et al. 2022; Zhou et al. 2019). For example, parenting training sessions were held during routine well‐child visits at 2 and 6 months of age by child development experts (China) (Zhou et al. 2019).

Only one of the interventions conducted in Tanzania was delivered remotely via weekly WhatsApp sessions lasting 1–2 h over a 6‐week period, during which facilitators hosted a group chat that provided an opportunity for questions and discussion (Skeen et al. 2023).

### 3.5 Target Population Characteristics

Thirteen of the interventions and programs were implemented in poor rural population where villagers were small‐scale farmers who worked in the forest or were involved in agricultural activities or in cattle rearing for their livelihood (Vietnam, Pakistan, China, Bangladesh, Armenia, India, Vietnam, Colombia, Kenya, Peru) (Attanasio et al. 2022; Baek et al. 2023; Bliznashka et al. 2022; Hossain et al. 2024; López García et al. 2021; Nuño et al. 2022; Rosales et al. 2019; Zhou et al. 2019). Seven of 34 studies targeted poor urban populations living in urban slums.

Included interventions and programs targeted children from birth to 2 years old and their primary caregiver. The age of the children targeted ranged from: 0 to 3 years old (Brazil, China) (Santos et al. 2022; Zhou et al. 2019); 6–24 months old and their primary caregivers (Kenya and Bangladesh) (López García et al. 2021; Tofail et al. 2018); 6–36 months old (Rwanda) (Desmond et al. 2023); 7–16 months old (India and Bangladesh) (Grantham‐McGregor et al. 2020; Hossain et al. 2022); and 9–32 months old (Tanzania) (Skeen et al. 2023). Three interventions targeted pregnant women, following them through pregnancy and their children for 2 years (Vietnam and India) (Baek et al. 2023; Gaidhane et al. 2023) or 18 months (Iran) (Bemanalizadeh et al. 2022). Similarly, three programs targeted pregnant women and their children up to 3 years of age (Brazil) (Buccini et al. 2021; Santos et al. 2022), 4 years old (Brazil) (Viegas Da Silva et al. 2022), and 5 years old (Tanzania) (Russell et al. 2022). In addition, two interventions targeted preschool children from 2 to 4 years old (India and Brazil, respectively) (Koshy et al. 2024; Weisleder et al. 2018).

### 3.6 Measured Outcomes: What Was Intended to be Improved?

The measured outcomes identified across the included studies were primarily intended to enhance cognitive, language, and motor development, aligned with opportunities for the early learning NCF domain. The remaining outcomes focused on good health, adequate nutrition, and responsive caregiving aiming to improve caregiver‐child interactions. The studies predominantly relied on standardized caregiver‐report scales, such as the Ages and Stages Questionnaire, third edition (ASQ‐3) (Desmond et al. 2023; Grantham‐McGregor et al. 2020; Jensen et al. 2021; Russell et al. 2022; Santos et al. 2022; Shi et al. 2020; Zhou et al. 2019), and direct observation instruments, such as the Bayley Scales of Infant and Toddler Development, third edition (BSID‐III) (Ahmed et al. 2023; Attanasio et al. 2022; Baek et al. 2023; Bemanalizadeh et al. 2022; Bliznashka et al. 2022; Hossain et al. 2022; López García et al. 2021; Luo et al. 2019; Nuño et al. 2022; Rosales et al. 2019; Sylvia et al. 2022), and the International Development and Early Learning Assessment (IDELA) (Pisani et al. 2016; Saori et al. 2019; Spier et al. 2020). Table 4 provides a comparative summary of the measurement instruments used across the included studies. Psychometric validity parameters of the scales were reported when available.

**Table 4:** Measurement instruments utilized in the included studies. Systematic review on early childhood development programs and interventions.

Instrument	Outcome	Target population	Psychometric characteristics	Psychometric limitations	Who administered
Ages and Stages Questionnaire, third edition (ASQ‐3) (Ages & Stages Questionnaire n.d)	Fine and gross motor coordination, language, cognitive and personal‐social development	Children aged 1–66 months	Reliability: > 0.92 Validity: Sensitivity: 86%, Specificity: 85% Note: Available in 40 languages.	‐No developmental cut‐offs have been validated for use in Rwanda (Jensen et al. (2021))	Trained field workers
Bayley Scales of Infant and Toddler Development, third edition (BSID‐III) (Balasundaram and Avulakunta 2026)	Cognitive, language and motor development.	Children aged 16 days to 42 months	N.R.	‐BSID assessment is time‐consuming and requires appropriate training. ‐Context validation required (Hossain et al. (2024))	Trained child development specialists
International Development and Early Learning Assessment (IDELA) (Pisani et al. 2018)	Literacy, numeracy, social‐emotional development, and motor skills.	Children aged 3.5–6 years	Reliability: 0.90	‐Requires trainee workers ‐Can be affected by the child's temperament	Trained field worker
Extended Ages and Stages Questionnaire (EASQ) (Ages & Stages Questionnaire n.d)	Communication, gross motor, and personal social development	Children aged 1–66 months	Reliability: > 0.92 Validity: Sensitivity: 86%, Specificity: 85% Note: Available in 40 languages.	‐Creation of internal standardization (Tofail et al. (2018))	Trained field workers
Wechler Preschool Primary Scale of Intelligence (WPPSI) (Gould et al. 2011)	Cognitive development	Children aged 2 years 6 months to 7 years 7 months	N.R.	‐Need to be applied by qualified professionals and training is required	Trained professionals
Malin's intelligence scale for Indian children (MISIC) (Koshy et al. 2024)	Cognitive development	Children aged 6–15 years 11 months	N.R.	‐Need to be applied by qualified professionals and training is required Note: is an adaptation of the Wechsler Intelligence Scale for Children (WISC)	Trained professionals
Developmental Milestones Checklist (DMC) (Gaidhane et al. 2023)	Motor, language, personal‐social, and emotional development	Children aged 3–24 months	Inter‐rater reliability: 0.875	N.R.	Trained field workers
Profile of Socio‐Emotional Development (PSED) (Gaidhane et al. 2023)	Social and emotional development	Children aged 0–5 years	Inter‐rater reliability: 0.673	‐Need adaptation to local context and incorporated cultural and socially relevant items.	Trained professionals
Early Childhood Development Index (ECDI) (UNICEF 2023)	Child development index ‐Literacy and numeracy skills, readiness to learn, physical growth, and social‐emotional development.	Children aged 24–59 months	‐Has been validated across various low‐ and middle‐income countries ‐Easy to understand and quick implementation	Caregiver response bias Lack sensitivity to cultural differences	Trained field workers
Caregiver Reported Early Development Index (CREDI) (Y. Li et al. 2020)	Child cognitive, language, and socio‐emotional development	Children aged 0–36 months	‐Has been validated across various low‐ and middle‐income countries	Caregiver response bias Population‐level assessment not at individual level	Trained field workers
Malawi Development Assessment Tool (MDAT) (Gladstone et al. 2010; Jensen et al. 2021)	Child motor, language, cognitive and social development	Children aged 0–60 months	Validity: Sensitivity: 97%, Specificity: 82% Developed to be culturally appropriate for use in rural Africa	N. R.	Trained professionals
INTERGROWTH‐21st Neurodevelopment Assessment (INTER‐NDA) (Waechter et al. 2022)	Cognition, motor, language, and behavior	Children aged 22–30 months	N. R.	Require specific training	Trained field workers
Peruvian Infant Development Scale (ESDI) (Nuño et al. 2024)	Child socio‐emotional, fine and gross motor, communication, and cognitive skills and an overall performance.	Children aged 0–36 months	Has been validated with the Screening Inventory of Battelle's Developmental Inventory (BDI) Inter‐rate reliability: 0.8	Require specific training	Trained field workers
Battelle's Developmental Inventory (BDI) (Berls and McEwen 1999)	Child five neurodevelopment domains: personal‐social, adaptive, motor, communication and cognitive.	Children aged 0–8 years	For age 4 years BDI instrument presents good validity for predicting later development. Inter‐rate reliability: 0.9 Sensitivity: 75%–90% Specificity: 80%–95%	Require specific training	Trained field workers
Integral child development: questionnaires to the children's mothers adapted from the UNICEF Multiple Indicator Cluster Survey (MICS) (UNICEF 2020)	Cognitive and sensorial stimulation	Children aged 0–12 months	‐Strong content validity because its indicators are aligned with global frameworks. Sensitivity: 70%–90% Internal consistency: 0.5–0.7	Require specific training	Trained field workers
StimQ (Dreyer et al. 1996)	Cognitive home environment and stimulation	Children aged 5 months to 6 years	Supported by strong evidence of validity and reliability, backed by multiple empirical studies. Good internal consistency, StimQ‐Infant: Cronbach's *α* ≈ 0.85	Require specific training	Trained field workers
Teste de Vocabulário por Imagens Peabody (TVIP) (Eigsti 2017)	Receptive vocabulary	Children aged 2.5 years and older	Easy to apply, doesn't rely on expressive language Inter‐rate reliability:1.0 Internal consistency: 0.9 Strong validity.	It can be influenced by contextual factors.	Trained field workers
Teste Infantil de Nomeação (Seabra and Dias 2012)	Child's expressive vocabulary	Children aged 2–6 years	Inter‐rate reliability: 0.85–0.95 Internal consistency: alfa = 0.7–0.9 Validity: 0.6–0.8	Partial view of language development	Trained field workers
Teste Infantil de Memória de Trabalho (TIMT) (Morais and Macedo 2011)	Working memory and cognitive development	Children from 4–12 years	The test shows solid construct validity, as tasks (e.g., digit span, sequencing) align with theoretical models of working memory (phonological loop, visuospatial sketchpad, central executive).	It assesses working memory specifically, not broader cognitive abilities. Performance may not fully reflect overall intelligence or learning capacity.	Trained field workers
Snijders‐Oomen nonverbal intelligence test (SON‐R) (Tellegen et al. 1998)	Non‐verbal cognitive abilities	Children aged 2.5–7 years	High internal consistency α: 0.90 Total score reliability: 0.9–0.93	Focuses exclusively on nonverbal intelligence. It does not assess vocabulary, verbal reasoning, language comprehension.	Trained field workers
Integrated Management of Childhood Illness (IMCI) (WHO n.d)	To assess, classify, and manage the health status of children under five years of age	Children under five years of age	NR	Does not measure a single construct. Instead, it provides a comprehensive clinical assessment framework to evaluate: Child morbidity, severity of illness, preventive care status. To guide decision‐making and treatment, rather than to generate a psychometric score.	Trained field workers
Home Measurement for Observation for the Environment (HOME) (Totsika and Sylva 2004)	Quality and quantity of stimulation and support available to a child in their home environment. Gross motor milestones. Responsive parenting.	Children aged 0–3 years (Infant/toddler HOME) Children aged 3–6 years (Childhood HOME)	Inter‐class correlation: 0.7–0.9	Cultural sensitivity, contextual and socioeconomic bias.	Trained field workers
Observation of the Mother–Child Interaction (OMCI) (Rasheed and Yousafzai 2015)	Quality of mother‐child interaction.	Children aged 6–36 months	Inter‐rater reliability: 0.691	Scoring depends on evaluators' judgment.Caregivers may change their behavior because they are being observed. Short observation.	Trained field workers

Abbreviation: NR, Not Reported.

### 3.7 Effects of Interventions and Programs

A substantial number of the included interventions and programs demonstrated significant positive effects on multiple ECD outcomes across different geographical contexts (*n* = 22), although the programs or interventions were heterogeneous in terms of design, NCF domains targeted, and contexts where they were implemented.

#### 3.7.1 Effects of Interventions Included

Interventions focused on the early learning NCF domain showed varying effectiveness based on the delivery method. In Tanzania, a group‐based parental intervention found significant improvements in cognitive and language development (0.22 SD), primarily due to increased stimulation at home and a reduction in punitive disciplinary practices (Leighton et al. 2023). Conversely, findings from China showed that parenting center‐based intervention achieved significantly smaller average impacts on child developmental outcomes and caregiver investment compared to home‐based alternatives (Sylvia et al. 2022). An intervention in India combined both home visits and group sessions, yielded significant developmental gains in cognition (0.324 vs 0.281 SD) and language (0.239 vs 0.302 SD), with all p values < 0.009; nevertheless, neither the home‐visits nor the group‐sessions demonstrated a superior average impact over the 2‐year follow‐up (Grantham‐McGregor et al. 2020).

Furthermore, the frequency of implementation moderated the intervention outcomes. In rural Peru, the combination of two home‐based interventions, comprising an Integrated Home‐Environmental Intervention Package (IHIP) (including kitchen sink, hygiene education, and a certified improved biomass cookstove), and an ECD component improved all domains of the Peruvian Infant Development Scale (ESDI). The pronounced effects were in the group receiving weekly ECD visits compared to the IHIP‐only group receiving biweekly visits. The ECD group showed higher odds in the cognitive domain (OR: 1.9; 95% CI: 1.1–3.5) and in overall performance (OR: 2.8; 95% CI: 1.6–4.9), reducing the risk of motor and language delay (Nuño et al. 2022). A home‐based intervention in China showed that children who received home visits more than once every 2 months had lower levels of suspected developmental delay than children in the control group (OR = 0.34, 95% CI: 0.18, 0.68) (M. Li et al. 2024). In this study, a higher frequency of home visits was associated with improved early stimulation by caregivers, the availability of children's books, and parent–child communication (M. Li et al. 2024). Similarly, evidence also from China identified a dose‐response relationship between frequency of delivery of ECD and clinic‐based programs or interventions and improved Ages & Stages Questionnaire (ASQ) scores (Zhou et al. 2019).

Interventions focused on the early learning NCF domain provided in preschool setting (*n* = 4) were also found to have a positive and lasting impact on children's literacy, numeracy, social, physical, and emotional development in India, Bangladesh, and Rwanda (Koshy et al. 2024; Saori et al. 2019; Spier et al. 2020). In Bangladesh, the intervention targeted 4‐year‐old children and was measured by the IDELA tool, which found significant improvement on literacy (0.23 SD), numeracy (0.30 SD), and social and emotional development (0.34 SD) (Spier et al. 2020). Similarly, in Rwanda, an intervention had the largest impact on emergent literacy and numeracy as measured by the IDELA tool, with older children scoring higher than younger children (Saori et al. 2019). In addition, evidence from India, indicated that structured early childhood education (ECE) with a duration of 18–24 months was significantly associated with higher cognition scores, specifically for processing speed showing the highest increase (β: 19.55 (11.26–27.77)), followed by full‐scale intelligence (β: 6.75 (2.96–10.550)) (Koshy et al. 2024).

Responsive stimulation and enhanced nutrition interventions from the Pakistan Early Child Development Scale‐up (PEDS) trial, led to significant improvement in cognitive, language, and motor scores at 24 months of age (Bliznashka et al. 2022). Two interventions combined, psychosocial stimulation (PS) and unconditional cash transfers (UCT), showed positive outcomes in rural and urban settings. In the rural context, children receiving the combined intervention showed significantly higher cognitive (B = 2.96, *p* = 0.021, ES = 0.24) and language scores (B = 2.73, *p* = 0.022, ES = 0.21) compared to the UCT‐only group (Hossain et al. 2022). Similarly, the intervened children in the urban context experienced improvements in cognitive [ES: 0.42 SD (95% CI: 0.58–0.25)], language [(0.38 SD, 95% CI: 0.55–0.22)], and motor [(0.17 SD, 95% CI: 0.01–0.34)] development (Hossain et al. 2024).

Regarding the adequate nutrition domain, two interventions reported nutritional outcomes, specifically improvement in diversity dietary scores (Bliznashka et al. 2022) and a more than two‐fold increase in the diversity of foods consumed by participating children (González‐Fernández et al. 2020). In addition, a multi‐dimensional intervention addressing all five domains of NCF, comprising home and community gardens, nutritional education, and parenting workshops bi‐weekly, by González‐Fernández et al. (2020), demonstrated a reduction in the risk of food insecurity (ARR = 0.20 (95% CI: 0.08–0.51)) and language delay (0.39 (0.19–0.82)). Similarly, an intervention in Armenia showed that parents were 55% more likely to provide minimum dietary diversity compared to control groups (aOR = 1.55, 95% CI: 1.10–2.19, *p* = 0.013) (Rosales et al. 2019). Furthermore, evidence regarding malnutrition outcomes reported no significant impact (Bliznashka et al. 2022; Hossain et al. 2022, 2024; Luo et al. 2019; Rosales et al. 2019). While in India, a statistically significant effect on weight‐for‐height (WHZ) was identified at 24 months for children in the intervention group (−1.47 (1.13; −1.60 −1.34), *p* = 0.033), but these effects did not extend to weight‐for‐age (WAZ) or height‐for‐age (HAZ) (Gaidhane et al. 2023).

Regarding the safety and security domain, in Rwanda, families receiving intervention decreased the use of harsh discipline (IRR = 0.741, 95% CI 0.657–0.835), and female caregivers showed a greater decrease in intimate partner violence (IRR = 0.616, 95% CI 0.458–0.828) (Jensen et al. 2021). Similarly, in Bangladesh, mothers in the intervention groups experienced less violence [OR; 0.6 (95% CI: 0.4–1.0)] and fathers engaged more (0.23 SD, CI: 0.39–0.06) in ECD activities (Hossain et al. 2024). In contrast, one intervention in Grenada did not find differences in corporal punishment beliefs, attitudes, and behaviors (Waechter et al. 2022).

#### 3.7.2 Effects of Programs Included

Regarding programs included, most of them were focused on early learning NCF domain. In this regard, evidence from the Brazilian program Primeira Infância Melhor (PIM) showed that starting the program during pregnancy was associated with 0.19 SD (95% CI −0.02–0.40) increase in development scores at age 4, and a 60% reduction in the prevalence of low developmental score (PR = 0.40; 95% CI 0.18–0.89) (Viegas Da Silva et al. 2022). In contrast, there was no strong statistical evidence of association with child outcomes for the PIM program starting after birth (Viegas Da Silva et al. 2022).

Based on a cross‐sectional analysis in Lao PR, one preschool program was significantly associated with improved development outcomes (Kamiya and Nomura 2023). Children who attended preschool had higher odds for literacy‐numeracy (OR = 4.754, *p* < 0.01). In addition, positive associations were observed for learning (OR = 2.009, *p* < 0.01), physical growth (OR = 3.867, *p* < 0.01), and social‐emotional development (OR = 1.188, *p* < 0.1) (Kamiya and Nomura 2023).

Furthermore, in Tanzania, a cross‐sectional analysis of a national ECD program, found that caregivers engagement was significantly associated with child development. Specifically, each additional caregiver‐child activity was associated with a 0.036 point increase in overall CREDI score (cognitive, language, social‐emotional, and motor) (95% CI = 0.014, 0.058, *p* = 0.002) (Russell et al. 2022).

Finally, regarding safety and security, an evaluation of a program in Brazil found no significant differences among control and intervention groups on responsive caregiving, disciplinary methods, or psychological attributes (Santos et al. 2022).

### 3.8 Cost Analysis and Synthesis of Cost‐Effectiveness Evidence

The main characteristics of the 10 interventions that reported cost‐effectiveness results are presented in Table 5. The intervention duration ranged from 3 to 24 months, with an average of 15.4 months. The number of children participating at baseline ranged from 168 to 2289, with an average of 1141 children. The reported cost per child participating at baseline ranged from $38 to $456, with an average of $197.4 per child.

**Table 5:** Results of cost‐effectiveness analysis in the studies. Systematic review on early childhood development programs and interventions.

		Baek et al.	Ahmed et al.	Lopez Garcia et al.	Waechter et al.	Leighton et al.	Desmond et al.	Attanasio et al.	Grantham‐McGregor et al.	Shi et al.	Spier et al.
Duration (months)		18	18	9	24	24	3	10.4	24	12	12
Number of participants		1245	511	1070	333	2289	1078	1460	1401	168	1856
Addressed domains of the Nurturing Care Framework	Good health	Yes	Yes	Yes	Yes	Yes	Yes	Yes	Yes	Yes	
	Adequate nutrition		Yes					Yes	Yes		
	Responsive caregiving	Yes			Yes	Yes	Yes	Yes	Yes	Yes	
	Opportunities for early learning		Yes	Yes	Yes	Yes		Yes	Yes	Yes	Yes
	Security and safety	Yes					Yes				
	Total domains	3	3	2	3	3	3	4	4	3	1
Costs	Perspective	The service provider and household	Healthcare provider	Societal	Provider	Societal	Provider	Provider	Provider	Primary health care institution provider	Societal
	Reported Costs (USD/child)	273	266	140	102	168‐329	456	333	38	51	145
	Year of costs	2019	2014	2020	2022^a^	2023^a^	2023^a^	2016	2020^a^	2014	2019
	Present‐value costs (2023)	350	380	191	108	266‐520	510	486	50	61	213
Cost‐effectiveness	Method of outcome evaluation^c^	Bayley III	Bayley III	Bayley III	INTER‐NDA	CREDI	ASQ‐3	Bayley III	Bayley III	ASQ‐C‐3	IDELA
	ICER (USD)	68	16	270^b^	472^b^	47–113^b^	4145^b^	2042^b^	NA	203^b^	1148^b^
	Conclusion	Cost‐effective	Cost‐effective	Cost‐effective	Cost‐effective	Cost‐effective	Cost‐effective	Cost‐effective	Effective (group sessions)	Cost‐effective	Cost‐effective

*Notes:* All the studies had child cognitive development as their primary outcome, except for Shi et al. (child development). Several of the studies had other primary and secondary outcomes. Leighton et al. reported results in pounds, we converted them to US dollars adjusted for purchasing power parity.

^a^ Based on the year of publication of the study.

^b^ Based on standardized score of the raw version.

^c^ Bayley III: Bayley Scales of Infant and Toddler Development, Third Edition; INTER‐NDA: The INTERGROWTH‐21st Neurodevelopment Assessment; CREDI: Caregiver Reported Child Development Instrument; ASQ‐3: Ages & Stages Questionnaires, Third Edition: Ages & Stages Questionnaires, Third Edition; ASQ‐C‐3: The Chinese version of the Ages & Stages Questionnaires‐ third edition; IDELA: International Development and Early Learning Assessment.

All interventions were reported as cost‐effective by the original studies; however, there is notable heterogeneity in the NCF domains covered, as well as in various technical aspects of the cost‐effectiveness estimation, such as the perspective, the target outcomes, duration, comparator, and even the method used to assess the main outcome (child cognitive development). The study by Grantham‐McGregor et al. (2020) was not strictly a cost‐effectiveness analysis of an ECD intervention; rather, it was a study that compared the effectiveness of two delivery mechanisms. The interventions studied by Leighton et al. (2023) and Shi et al. (2020) stand out among the interventions that reported their results in standardized units of raw scores, as they have the lowest reported ICERs and address three of the five dimensions of the NCF.

### 3.9 RE‐Aim Analysis

The detailed RE‐AIM analysis can be found in Supporting Information 4 and is summarized below.

The RE‐AIM analysis included those studies with cost information and implementation data (*n* = 5). Related to the analysis, in the Reach dimension, all included studies reported on the description of the target population, methods of identifying the target population, and inclusion criteria. However, none of the studies reported the participation rate or coverage. The target population was slightly different, ranging from children aged 1–2 months and their caregivers; mothers with their children aged 6–24 months or mothers with children aged 7–16 months; children aged 0–24 months; and children aged 4 years.

All the studies analyzed reported measures/results within the effectiveness dimension. However, only three studies (60%) reported economic impact, and none reported on quality of life or potential adverse outcomes.

In terms of Adoption, the level of the targeting intervention was reported in all the analyzed studies, but the extent of adoption of the intervention across settings was not reported in any study. Important factors to adoption were reported in three studies (80%), such as collaboration with non‐governmental organizations for implementation (López García et al. 2021; Spier et al. 2020) and incorporating the intervention into a pre‐existing program (Attanasio et al. 2022). Another important factor for adoption was related to limited resources in terms of cost, infrastructure, and adequately trained human resources (Shi et al. 2020; Spier et al. 2020).

Regarding Implementation, on the one hand, the adaptations made to interventions reported were related to the challenges faced, such as logistical difficulties in delivering materials and nutritional supplements in complicated rural geographies. There was a short duration of participant exposure to the programs and marked heterogeneity in the fidelity of the intervention's implementation. The resistance of providers to change their behavior was also reported as a challenge at the initial stages of implementation (Attanasio et al. 2022). Moreover, non‐attendance of target participants emerged as a significant barrier with temporary migration affecting participation in home visiting and nutritional education, and mothers' time and other constraints limiting attendance at group sessions (Grantham‐McGregor et al. 2020). On the other hand, enabling factors included investments in training local trainers and delivery agents and regular supervision of intervention delivery (López García et al. 2021; Spier et al. 2020).

In relation to Maintenance, two studies reported enabling factors for scalability, such as the integration of the intervention into existing services (established infrastructure facilitates access and compliance among participants) and training of healthcare workers (reduces the costs associated with capacity‐building) (Attanasio et al. 2022; Shi et al. 2020).

## 4 Discussion

In this systematic review, we examined and characterized early childhood development (ECD) interventions and programs implemented in LMICs, using the Nurturing Care Framework (NCF) and the RE‐AIM model to assess their effects, implementation challenges, scalability, and cost‐effectiveness. Despite strong evidence on the key NCF domains, our findings highlight that most studies focused on early learning, health and nutrition, while other critical domains of the NCF such as safety and security remained insufficiently addressed. We identified a wide variation in delivery strategies, intensity, and targeted populations across studies, together with moderate methodological quality and reporting limitations, complicating direct comparison across studies and the interpretation of their effects. Our study contributes to the existing knowledge by systematically identifying the various interventions and programs aimed at improving ECD in LMICs. It adds to the existing literature through an in‐depth analysis of the geographic distribution of published evidence, the domains of the NCF addressed in the programs and interventions, and the methods used to deliver the components of these actions. Additionally, we identified implementation challenges and its enablers, analyzed the associated implementation costs, and assessed their cost‐effectiveness. Differences between interventions and programs, as defined in this review (i.e., time. bound actions vs. structures, long‐term strategies with broader reach and funded with government resources), may reflect variations in implementation context and sustainability, which are important considerations when interpreting their potential impact at scale.

We identified numerous studies evaluating interventions and programs to improve ECD, with many randomized trials showing moderate to adequate quality. However, key methodological weaknesses persist, including unclear group assignment methods, insufficient intervention descriptions, unreported contamination risks, and limited population characterization. Addressing these gaps is essential to strengthening the evidence in this field. Most of the data came from countries in Africa and Asia. In contrast, there is a notable lack of available information from the Americas.

Our findings suggest that most interventions and programs remain fragmented in their design and focused on selected NCF domains rather than adopting a fully integrated approach. The Integrated Early Childhood Development (IECD) program in rural China, targeting poverty‐stricken areas, is one example that incorporates all five components (Zhou et al. 2019). Importantly, Zhou et al. (2019) reported barriers related to the weak service capacity in poor rural areas of China, including integrating multiple services into an existing intervention.

Among all the interventions and programs included in this review, the safety and security domain is the least addressed. The Happy Child Program (Criança Feliz Program ‐CFP‐) in Brazil includes this domain by incorporating it into its objectives which are guiding and supporting vulnerable pregnant women and their families in preparation for the childbirth, collaborating in the exercise of parenting, strengthening the roles of families in the care, protection and education of children and favoring the strengthening of affective and community bonds as well as promoting actions for integral development in early childhood (Buccini et al. 2023).

The types of interventions and delivery methods were highly varied, ranging from those directly focusing on ECD to those focusing on improving hygiene, food security, child nutrition, responsive childcare, and partner violence. The most common delivery setting and method was the health sector via group sessions and home visits by community workers. Moving forward, it is important for other NCF domains to be addressed, such as safety and security, with the First Read program being an example. Technology has been widely used for program delivery in various fields, including mHealth (DeWitt et al. 2022). This review identified only one intervention, implemented in Tanzania (Skeen et al. 2023), where group sessions were conducted via WhatsApp. Using such technologies, if proven effective, can enhance attendance and adherence to the interventions at a reduced cost.

The target population varied widely in terms of children's age, with most interventions focusing on children under 2 years old, while only a few began during pregnancy or continued until 2–5 years of age. This is worth highlighting given the importance of promoting ECD through a life course perspective, starting from the preconceptional stage, followed by the prenatal stage and extending into preschool and school years (Black et al. 2017; Daelmans et al. 2021). There were very few studies focusing on preschool program implementation, which is a major gap because of the importance of the preschool period for child development (Draper et al. 2024; Nores et al. 2024).

Most of the programs and interventions targeted poor children living in rural areas, which is important from an equity perspective. However, considering the growing rates of urban poverty, especially in periurban areas, it is essential to fill this key knowledge gap.

Most of the programs and interventions studied in the literature reviewed impacted ECD cognitive and socioemotional development. Some of the programs and interventions also had an impact on parenting and nutritional status. Furthermore, national programs acknowledged as being examples for the world to follow are consistent with our findings, especially the program Chile Crece Contigo (ChCC). Although it was not included in our review because it did not meet one of the selection criteria (programs or interventions in LMICs), we consider it important to mention it due to its success and impact, and geographical location. In addition, when the program was launched in 2007, Chile was classified as a middle‐income country, although now it is classified as a high‐income country (OECD 2020). ChCC supports children from conception to school entry, strongly emphasizing vulnerable populations. The program integrates universal services, such as prenatal care, parenting education, and child health monitoring, with targeted interventions for at‐risk children, including home visits and psychosocial support. Through a multisectoral and integrated approach, ChCC coordinates efforts across the health, education, and social protection sectors, ensuring continuity of care through personalized follow‐ups and case management. It also promotes family‐centered support, encouraging parental engagement in workshops on responsive caregiving, early stimulation, and parenting skills while providing psychosocial assistance to vulnerable families. ChCC's evidence‐based strategies have demonstrated positive outcomes in child health, nutrition, and cognitive development. Its success is driven by strong political commitment, a focus on equity and children's rights, and sustained government investment (Torres et al. 2018).

As enablers for adoption and implementation of intervention, the availability of financial, infrastructure, and adequately trained personnel resources were highlighted as key enablers, a finding consistent with previous evidence (Black et al. 2017; Buccini et al. 2021; Cavallera et al. 2019; Coore‐Hall et al. 2023; Khan et al. 2018; Milman et al. 2018; Pérez‐Escamilla et al. 2018; Rao and Kaul 2018; Richter et al. 2017; Richter and Samuels 2018; Torres et al. 2018). However, in our review, few studies were identified with data about the quality of the training received by the field team. In addition, due to the facility of access to the target population and cost reduction, a key enabler was identified as the integration of the intervention or program into an existing service through an established infrastructure, with implications for scalability, as has been reported by others (Britto et al. 2018; Buccini et al. 2021; Richter et al. 2017).

Even though evidence indicates that stakeholder engagement is key for effective adaptation, implementation, and scalability of ECD programs and interventions (Coore‐Hall et al. 2023), none of the studies included in our review reported information on stakeholder involvement. In addition, our findings highlight important gaps across RE‐AIM dimensions, including limited reporting on population reach (i.e. coverage), lack of evidence on adoption across settings, and scarce information on maintenance and long‐term sustainability. Finally, variability in implementation fidelity and the need for context‐specific adaptations underscore the dynamic and context‐dependent nature of scaling up and sustaining ECD interventions.

Regarding the cost‐effectiveness analysis, we identified only 10 studies containing cost information. These studies displayed a wide range of outcomes, dimensions, durations, intervention intensities, and comparators, making it difficult to assess relative cost‐effectiveness. To address this gap, we performed a narrative review identifying their main characteristics and estimating costs in constant 2023 values to facilitate comparability, highlighting two interventions for covering three domains of the NCF while maintaining relatively low ICERs. However, more cost‐effectiveness studies are urgently needed to support effective decision‐making in the field.

Although most studies were rated as moderate to high quality, common methodological limitations, such as insufficient reporting of intervention components, unclear group allocation, and limited population characterization, may have biased the observed effect and their generalizability.

One of the strengths of our review is that it was guided by the global evidence‐based NCF. Additionally, to our knowledge, this is the first review to include a cost‐effectiveness analysis, presenting a narrative synthesis of the results. Finally, our analysis includes a comprehensive review of various aspects (e.g., components of the NCF, target population, combinations of actions, delivery platforms, among others) of interventions and programs implemented in LMICs. Our review has some limitations that should be considered. One of them, common in any type of review, is publication bias, which could have excluded studies with negative or null results on our outcome variables, potentially biasing our findings. However, in our review, we identified studies reporting small or null effects of the interventions. Another limitation is the quality of the included studies, which could affect the reliability of our results and conclusions. However, more than 80% of the included studies had moderate or adequate quality in terms of internal and external validity.

## 5 Conclusions and Recommendations

This review shows that few ECD interventions and programs fully integrate all domains of the NCF, despite the importance of doing so to achieve meaningful and sustained impact. While existing evidence provides valuable insights into key implementation challenges and enablers, important gaps remain in understanding how to effectively scale and sustain these interventions across diverse contexts. Strengthening the empirical evidence on implementation processes and cost‐effectiveness of ECD interventions and programs will be critical to inform policy and guide decision‐making for the expansion of ECD programs in LMICs.

## Author Contributions

S.H.‐C., B.F.‐L. conceptualized the review and led the overall study design. S.H.‐C., B.F.‐L., M.H.‐F., V.L.‐M. and P.E.‐R. developed the search strategy and conducted the literature screening. Data extraction was performed and cross‐checked by S.H.‐C., B.F.‐L., V.L.‐M., M.P.‐C. S.H.‐C., B.F.‐L., M.H.‐F., V.L.‐M., M.P.‐C. conducted the quality appraisal and contributed to data synthesis. R.P.‐E. provided key conceptual input throughout the process and offered substantive feedback to strengthen the manuscript. S.H.‐C. and B.F.‐L. drafted the initial manuscript with input of all authors. All authors critically revised the manuscript and approved the final version.

## Conflicts of Interest

The authors declare no conflicts of interest.

## Supporting information


Supporting File 1

Supporting File 2

Supporting File 3

Supporting File 4

## Data Availability

The data that support the findings of this study are available from the corresponding author upon reasonable request.
