# Supplementary material for: Strengthening Early Childhood Development Programs and Interventions in Low‐ and Middle‐Income Countries: Systematic Review of Interventions
Source: Matern Child Nutr. 2026 Sep 1;22(3):e70244. doi: 10.1111/mcn.70244 (PMC13531360; doi:10.1111/mcn.70244)
Supplement: Supplementary file 1 [file MCN-22-e70244-s001.docx]

**Supplementary Table 4.** RE-AIM analysis ECD interventions.

1. **Key elements linked to an ECD intervention in China from a perspective of the RE-AIM.**

| **RE-AIM Element** | **ECD intervention in China: *Parenting intervention*** |
| --- | --- |
| **REACH** | |
| Description of target population (geographic coverage, numbers targeted, demographic characteristics) | Infants aged 1-2 months and their caregivers |
| Setting characteristics (urban vs. rural, etc.) | Urban communities (primary health care) |
| What percent of potentially eligible participants a) were excluded, b) took part and c) how representative were they? |  |
| Characteristics of participants reached compared to non-participants or to target population | Participants were all children and their caregivers in the communities who met the following inclusion criteria and were invited to participate in the study in February and June 2014: term infants aged 1-2 months with birth weights > 2500 g, and the caregiver’s agreement to participate in the study. |
| Other factors that affect the reach of the program, including policy context, program budget constraints, conflict, and cost. | Higher attrition rate in the control group due to family migration could introduce bias in evaluating the intervention's effects. |
| **EFFECTIVENESS** | |
| What were the goals of the program? | To provide a parenting intervention integrated with primary child health care regarding ECD. |
| What were the successes achieved toward stated goals of the program? | -*Child development outcomes*: children in the intervention group showed significantly higher scores in communication (adjusted mean difference = 0.26; 95% CI 0.03, 0.51), fine motor skills (adjusted mean difference = 0.19; 95% CI 0.01, 0.37), and overall development (adjusted mean difference = 0.25; 95% CI 0.10, 0.41) as measured by the Ages and Stages Questionnaire (ASQ-C) at 14 months of age compared to the control group. |
| What impact did the intervention have on all participants who began the program? | Improved child development, particularly communication and fine motor skills. |
| What was the effect on process intermediate, and primary outcomes? | The intervention had significant effects on child development at the age of 14 months, i.e., after 12 months of the intervention.  The children’s language and fine motor development benefited more from the intervention than did their cognitive and personal-social development. |
| Possible negative or unintended consequences of the intervention on quality of life and related factors |  |
| Economic impact | The intervention costs associated with increasing the communication, fine motor, and overall ASQ-C scores by one SD at 14 months of age were $195.65, $267.74, and $203.48, respectively |
| **ADOPTION** |  |
| What level was the program/intervention targeting (e.g., individual, community, policy)? How representative were the participating settings? | At the community level. But, the target is the caregivers and children within the community. |
| Description of regulations and legislation and how these affected adoption and continued use |  |
| Other factors important to adoption | *Resource Limitations:* Implementing similar ECD interventions in primary health care settings may require adequate space and sufficient child development specialists, which could be a challenge in low- and middle-income countries. |
| **IMPLEMENTATION** |  |
| Description of implementation strategy including underlying theory, if any, and how it may be integrated with any other interventions | The intervention consisted of a parenting program that included three main components:  1. Parenting Pamphlet: Caregivers received a pamphlet compiled by researchers that provided information on age-appropriate games and activities to support children's development in motor, communication, cognition, and social-emotional skills. The pamphlet was designed using simple Chinese language and included detailed descriptions of the benefits, methods, duration, and frequency of each activity. Was distributed at enrollment.  2. Parenting Training Sessions: Two 2-hour training sessions were held during routine well-child visits at 2 and 6 months of age. These sessions were conducted by child development experts and aimed to enhance caregivers' understanding and skills in supporting their children's development.  3. Additional Guidance: For caregivers of children suspected of having developmental delays, additional parenting guidance was provided via telephone to address specific concerns and further support their child's development.  The control group received routine primary health care services, which included regular physical examinations, disease screening, vaccinations, health, nutrition, and development consultations and guidance. |
| Cost of intervention (time or money) from the implementer perspective | The total intervention cost was $4171.32, and the cost per child from 2 to 14 months was $50.87. The intervention costs associated with increasing the communication, fine motor, and overall ASQ-C scores by one SD at 14 months of age were $195.65, $267.74, and $203.48, respectively. |
| Consistency of implementation across staff/time/settings/subgroups (not about differential outcomes, but process) |  |
| Adaptations made to intervention during program/project roll out (i.e., was the intervention delivered as intended?) | No important changes took place during implementation. |
| Other factors important to implementation, including policy and regulatory environment. |  |
| **MAINTENANCE** |  |
| To what extent were different intervention components continued or institutionalized? How was the original program modified? | The intervention lasted 12 months. |
| Availability/accessibility of intervention over time, and importance of these factors to adoption and sustained use | Authors reported enablers for the success of ECD intervention:  1. Integration with Existing Services: The intervention was delivered within the established infrastructure of primary health care services, facilitating access and compliance among caregivers.  2. Training of Health Care Workers: The existing literacy and training of primary health care workers reduced the costs associated with capacity-building for ECD interventions. This enabled health care providers to deliver the parenting program components effectively. |
| Is the intervention still ongoing at ≥ 12 months? | No, the intervention lasted 12 months. |
| Description of any long-term subsidies/incentives and plans for continuity or phase-out, and their effects on adoption/sustained use | None. |
| Reference:  Shi, H., Li, X., Fang, H., Zhang, J., & Wang, X. (2020). The Effectiveness and Cost-effectiveness of a Parenting Intervention Integrated with Primary Health Care on Early Childhood Development: a Cluster-Randomized Controlled Trial. Prevention science : the official journal of the Society for Prevention Research, 21(5), 661–671. https://doi.org/10.1007/s11121-020-01126-2 | |

2. **Key elements linked to ECD intervention in Kenya from a perspective of the RE-AIM**

| **RE-AIM Element** | **ECD intervention in Kenya: Msingi Bora (“Good Foundation” in Swahili) -a group-based parenting intervention-** |
| --- | --- |
| **REACH** | |
| Description of target population (geographic coverage, numbers targeted, demographic characteristics) | Mothers with children aged 6 to 24 months |
| Setting characteristics (urban vs. rural, etc.) | Rural setting |
| What percent of potentially eligible participants a) were excluded, b) took part and c) how representative were they? |  |
| Characteristics of participants reached compared to non-participants or to target population | Eligible participants within villages were mothers or other female primary caregivers aged 15 and over with a child between 6 and 24 months at recruitment without signs of severe mental or physical impairment. |
| Other factors that affect the reach of the program, including policy context, program budget constraints, conflict, and cost. |  |
| **EFFECTIVENESS** | |
| What were the goals of the program? | The Msingi Bora aimed to improve child cognitive, socioemotional development, and child receptive language |
| What were the successes achieved toward stated goals of the program? | -*Group-only arm:* The ITT effect sizes of improvements in the group-only arm were 0.52 standard deviation (SD; P = 0.001) in cognition, 0.42 SD (P = 0.017) in receptive language, and 0.23 SD (P = 0.024) in socioemotional development.  *-Mixed-delivery arm:* In the mixed-delivery arm, the ITT effect sizes were 0.34 SD (P = 0.021) in cognition, 0.20 SD (P = 0.17) in receptive language, and 0.22 SD (P = 0.011) in socioemotional development. Although the mixed-delivery arm had smaller effect sizes on child cognition and receptive language, differences across intervention arms were only statistically significant based on 1-sided tests at the 10% level. |
| What impact did the intervention have on all participants who began the program? | Significantly improved child cognitive, receptive language, and socioemotional development among treated families. |
| What was the effect on process intermediate, and primary outcomes? |  |
| Possible negative or unintended consequences of the intervention on quality of life and related factors |  |
| Economic impact | For the group-only delivery model an additional US$100 investment is estimated to lead to a 0.37 SD improvement in child cognition, 0.30 SD improvement in receptive language, and 0.16 SD improvement in socioemotional development. Under the mixed-delivery model, the corresponding estimates are improvements of 0.23 SD, 0.14 SD, and 0.15 SD, respectively. |
| **ADOPTION** |  |
| What level was the program/intervention targeting (e.g., individual, community, policy)? How representative were the participating settings? | Community level. |
| Description of regulations and legislation and how these affected adoption and continued use. | The intervention was conducted in collaboration with a Kenyan non-governmental organization (NGO), the Safe Water and AIDS Project (SWAP). |
| Other factors important to adoption | Parental attendance to the group sessions was an anticipated challenge in our dispersed and disadvantaged rural setting. Indeed, mothers with more education and who lived farther from a meeting place had lower attendance on average, suggesting an opportunity cost of attendance. However, the effect of distance was small: each additional kilometer to travel resulted in −0.7 fewer group sessions attended, and the median distance mothers had to travel was 0.65 kilometers. Although maintaining high attendance is an issue with many group programs, perceived benefits became a motivator. Until mothers saw the program's benefits, reminders and incentives were likely required, but mothers did not recognize their importance by the end of our program. |
| **IMPLEMENTATION** |  |
| Description of implementation strategy including underlying theory, if any, and how it may be integrated with any other interventions | *-Group-only delivery model:* 12 non-review group sessions held fortnightly and 4 group review sessions; monthly 1-day refresher training for CHVs was performed in each sub country for that month's sessions.  *-Mixed-delivery model:* 12 non-review group sessions held fortnightly and 4 review sessions delivered through home visits. In these home visits, CHVs delivered identical content to the group review sessions, but the focus was personalized on that family. CHVs visited each participant household during the same week that a group review session was held in group-only villages.  *-Control:* No sessions (only received a flyer on child feeding during the baseline survey that was made available to all respondents in all study arms). |
| Cost of intervention (time or money) from the implementer perspective | The total costs for the program were US$56,171 for the group-only arm and US$58,103 for the mixed-delivery arm, which translates into a total cost per child of US$140 in the group-only arm and US$145 in the mixed-delivery arm. |
| Consistency of implementation across staff/time/settings/subgroups (not about differential outcomes, but process) | Regarding implementation fidelity, CHVs’ experience delivering sessions and frequent supervisory feedback over the 8-month intervention led to significant improvements in CHV delivery performance and enjoyment of mothers and CHVs with the program. |
| Adaptations made to intervention during program/project roll out (i.e., was the intervention delivered as intended?) | No important changes took place during implementation. |
| Other factors important to implementation, including policy and regulatory environment. | An upfront investment in training local trainers and delivery agents, and regular supervision of delivery of a manualized program, appear key to our documented success. |
| **MAINTENANCE** |  |
| To what extent were different intervention components continued or institutionalized? How was the original program modified? | The intervention is still ongoing. |
| Availability/accessibility of intervention over time, and importance of these factors to adoption and sustained use | There is limited data about the impact of the intervention. |
| Is the intervention still ongoing at ≥ 12 months? | Yes, it is still ongoing. |
| Description of any long-term subsidies/incentives and plans for continuity or phase-out, and their effects on adoption/sustained use |  |
| Reference:  1.-Lopez Garcia I, Saya UY, Luoto JE. Cost-effectiveness and economic returns of group-based parenting interventions to promote early childhood development: Results from a randomized controlled trial in rural Kenya. PLoS Med. 2021 Sep 28;18(9):e1003746. doi: 10.1371/journal.pmed.1003746. PMID: 34582449; PMCID: PMC8478245.  2.-Luoto JE, Lopez Garcia I, Aboud FE, Singla DR, Zhu R, Otieno R, Alu E. An Implementation Evaluation of A Group-Based Parenting Intervention to Promote Early Childhood Development in Rural Kenya. Front Public Health. 2021 May 5;9:653106. doi: 10.3389/fpubh.2021.653106. PMID: 34026713; PMCID: PMC8131637. | |

3. **Key elements linked to ECD intervention in Bangladesh from a perspective of the RE-AIM**

| **RE-AIM Element** | **ECD in Bangladesh: *Early Years Preschool Program (EYPP)*** |
| --- | --- |
| **REACH** | |
| Description of target population (geographic coverage, numbers targeted, demographic characteristics) | Children 4 years old |
| Setting characteristics (urban vs. rural, etc.) | Rural setting |
| What percent of potentially eligible participants a) were excluded, b) took part and c) how representative were they? |  |
| Characteristics of participants reached compared to non-participants or to target population | In October 2017, authors conducted a census around all 100 schools to identify children who lived within a 15-minute walk of the school and were in the target age range—that is, children expected to enroll in typical government pre-primary in 2019 and enter Grade 1 in 2020.  Children selected for the study were invited to participate in the EYPP at their local school during the 2018 school year and then would go on to government pre-primary as usual in 2019. |
| Other factors that affect the reach of the program, including policy context, program budget constraints, conflict, and cost. |  |
| **EFFECTIVENESS** | |
| What were the goals of the program? | The EYPP aims to ensure holistic development for children and create early learning opportunities for younger children |
| What were the successes achieved toward stated goals of the program? | Positive intent-to-treat (ITT) impact of 0.23 standard deviations on children’s literacy, 0.30 standard deviations on numeracy, and 0.34 standard deviations on social and emotional development; these increases should be expected with scale-up of the EYPP. |
| What impact did the intervention have on all participants who began the program? | When compared to children’s natural progress when the EYPP is not available in their communities, on average, after the mandatory preschool year (i.e., in other words, the difference in midline scores and endline scores for children in the control group), an additional year of preschool was found to increase literacy, numeracy, and social emotional development scores by 18 percent, 26 percent and 38 percent, respectively. |
| What was the effect on process intermediate, and primary outcomes? | Positive impacts on children’s overall school readiness, beginning literacy, numeracy, and social and emotional development persisted from midline to endline. |
| Possible negative or unintended consequences of the intervention on quality of life and related factors |  |
| Economic impact | The midpoint cost estimate suggested a total annual cost of US$3,146 per community to provide the EYPP in the 50 intervention communities. |
| **ADOPTION** |  |
| What level was the program/intervention targeting (e.g., individual, community, policy)? How representative were the participating settings? | At community level. But, the target is 4 years old children. |
| Description of regulations and legislation and how these affected adoption and continued use | In 2013, Save the Children, along with representatives from the Ministry of Women and Children Affairs, DPE, under the Ministry of Primary and Mass Education, developed the EYPP to extend preschool education down to children age four. |
| Other factors important to adoption | Teachers focused on working conditions, specifically the need for a higher honorarium and more ongoing training (monthly).  EYPP teachers were very positive about the program, believing that it was beneficial for children.  Teachers mostly felt that the curriculum was appropriate but did report that the curriculum was somewhat less effective at teaching vocabulary and how the world works, and some of the mathematics lessons were too easy for many of the children. Children’s attendance at the EYPP (among those enrolled) was very high. |
| **IMPLEMENTATION** |  |
| Description of implementation strategy including underlying theory, if any, and how it may be integrated with any other interventions | The EYPP provides preschool education to children 4 years of age, offering younger children the possibility of receiving 2 years of preschool education instead of only 1 year (at age 5).  The EYPP aims to ensure holistic development for children and create early learning opportunities for younger children. By offering an additional year of preschool education, the EYPP is able to provide additional pre-primary learning experiences for children that are expected to translate into better outcomes, both in terms of school readiness and in terms of subsequent primary education outcomes, |
| Cost of intervention (time or money) from the implementer perspective | The midpoint cost estimate suggested a total annual cost of US$3,146 per community to provide the EYPP in the 50 intervention communities. Financial reports that involved expenditures per child indicate there were 1,084 beneficiaries, and thus the estimated total costs translate into an annual unit cost of $145 per child.  Note that these estimates capture the total costs of providing the EYPP (including things like use of extant classrooms, and school and community contributions to teacher’s salary), not just the expenditures by Save the Children. |
| Consistency of implementation across staff/time/settings/subgroups (not about differential outcomes, but process) |  |
| Adaptations made to intervention during program/project roll out (i.e., was the intervention delivered as intended?) | No important changes took place during implementation.  Based on the monitoring information available, the program was implemented with a high level of fidelity. Where classes had lower levels of fidelity, the issues tended to involve instructional time for children (e.g., class starting late and some attendance difficulties, although attendance issues were minimal overall). The monitors noted few concerns regarding program delivery. |
| Other factors important to implementation, including policy and regulatory environment. |  |
| **MAINTENANCE** |  |
| To what extent were different intervention components continued or institutionalized? How was the original program modified? | The intervention did not continue. |
| Availability/accessibility of intervention over time, and importance of these factors to adoption and sustained use |  |
| Is the intervention still ongoing at ≥ 12 months? | No, the intervention lasted one school year. |
| Description of any long-term subsidies/incentives and plans for continuity or phase-out, and their effects on adoption/sustained use | None |
| Reference:  1.-Spier, E., Rahman A., Nahar, Z. 2018. Bangladesh Early Years Preschool Program Impact Evaluation. https://thedocs.worldbank.org/en/doc/311791528140704115-0090022018/original/BangladeshECDbaseline.pdf  2.-Spier, E, Kamto, K, Molotsky, A, Rahman, A, Hossain, N, Nahar, Z. 2020. Bangladesh Early Years Preschool Program Impact Evaluation. Endline Report for the World Bank Strategic Impact Evaluation Fund. https://thedocs.worldbank.org/en/doc/954671593519042614-0090022020/original/2011381AIREYPPEndlineReport23April2020.pdf | |

4. **Key elements linked to ECD intervention in Colombia from a perspective of the RE-AIM**

| **RE-AIM Element** | **ECD intervention in Colombia: Family, “Women, and Childhood program” (FAMI for its acronym in Spanish) -parenting intervention-** |
| --- | --- |
| **REACH** | |
| Description of target population (geographic coverage, numbers targeted, demographic characteristics) | Children aged 0 to 24 months |
| Setting characteristics (urban vs. rural, etc.) | The study towns were located in three departments in central Colombia (Cundinamarca, Boyaca, and Santander). |
| What percent of potentially eligible participants a) were excluded, b) took part and c) how representative were they? |  |
| Characteristics of participants reached compared to non-participants or to target population | The departments were all chosen to have (i) fewer than 40,000 inhabitants, to avoid large urban centers, (ii) at least two FAMI units.  Eligible participants within the department were children under 12 months. |
| Other factors that affect the reach of the program, including policy context, program budget constraints, conflict, and cost. |  |
| **EFFECTIVENESS** | |
| What were the goals of the program? | To enhance the existing program through three  complementary elements:  (i) a structured early stimulation curriculum to improve child development, accompanied by pedagogical materials such as books, puzzles, and toys;  (ii) training and coaching for the FAMI mothers; and,  (iii) a larger and higher quality nutritional supplement than that previously received by FAMI participants, along with nutrition education during group sessions and home visits, and other materials such as recipe books and cards with age appropriate nutrition messages. |
| What were the successes achieved toward stated goals of the program? | The effect of the program on the Bayley-III factor was 0.163 SD, and it is statistically significant at 5%.  The fraction of children whose height-for-age was below -1 SD decreased by 6.8 percentage points or 0.15 SD, while the number of children with normal height-for-age increased by a similar fraction (7.6 percentage points) (p<0.05).  No significant average impact of the program on socio-emotional development or height-for-age Z-scores was found.  Do not find any statistically significant program effects on maternal knowledge about child development, maternal self-efficacy, or food insecurity. |
| What impact did the intervention have on all participants who began the program? | Significantly improved child development among treated families. |
| What was the effect on process intermediate, and primary outcomes? |  |
| Possible negative or unintended consequences of the intervention on quality of life and related factors |  |
| Economic impact |  |
| **ADOPTION** |  |
| What level was the program/intervention targeting (e.g., individual, community, policy)? How representative were the participating settings? | Community level. |
| Description of regulations and legislation and how these affected adoption and continued use. | The intervention was added to an existing government group based parenting support intervention, combined with nutritional supplementation. |
| Other factors important to adoption |  |
| **IMPLEMENTATION** |  |
| Description of implementation strategy including underlying theory, if any, and how it may be integrated with any other interventions | The FAMI program is delivered through weekly group sessions of one hour each and a monthly home visit of about an hour for parents of children 0–24 months of age. FAMI mothers, the program facilitators, are local women and generally have a high school degree but no specific training on ECD. The monthly home visits were not designed around stimulation activities for the child but involved general advice for the family. The program also delivers a nutritional supplement that corresponds to 22%–27% of the (monthly) recommended calorie intake of children younger than two and pregnant women.  *Intervention group* to enhance the existing program through three elements:  (i) a structured early stimulation curriculum to improve child development, accompanied by pedagogical materials such as books, puzzles, and toys; (ii) training and coaching for the FAMI mothers; and (iii) a larger and higher quality nutritional supplement than that previously received by FAMI participants, along with nutrition education during group sessions and home visits, and other materials such as recipe books and cards with age appropriate nutrition messages. The stimulation curriculum includes a monthly home visit. Both group meetings and home visits had a duration for about an hour.  The intervention also included a monthly nutritional supplement that provided 35% of the daily calorie intake requirements for target children. All supplements were delivered monthly to the FAMI facilitator, who was in charge of distributing them among program participants during the first group session of each month. |
| Cost of intervention (time or money) from the implementer perspective | Financial cost of the intervention: the cost of the pedagogical component of the intervention was equation $115 US per child per year plus an equation $11 US one-off cost per child for FAMI pre-service training.  The total increase in the cost of the program is of $322 US (or $333 US adding the one-off initial training), which effectively amounts to doubling the original cost of $327 US per child per year. |
| Consistency of implementation across staff/time/settings/subgroups (not about differential outcomes, but process) |  |
| Adaptations made to intervention during program/project roll out (i.e., was the intervention delivered as intended?) | It was not possible to fully control and enforce the many relevant implementation aspects that might be needed to ensure fidelity of the intervention and impact development.  Implementation of the intervention faced various challenges: low duration of participant exposure to the program, logistical difficulties for the delivery of pedagogical materials and the nutritional supplements in complicated rural geographies, heterogeneity in the fidelity of program implementation, and initial resistance of program providers to change their behavior. |
| Other factors important to implementation, including policy and regulatory environment. |  |
| **MAINTENANCE** |  |
| To what extent were different intervention components continued or institutionalized? How was the original program modified? | The intervention lasted 15 months approximately However, the FAMI program is still ongoing. |
| Availability/accessibility of intervention over time, and importance of these factors to adoption and sustained use | The authors pointed out that the design offers a directly scalable policy, both in terms of its costs and in its implementability, given the existing infrastructure and human resources.  The focus on the scalability is one of the most salient aspects of this study and reflects the difficulties policy makers face when moving from small trials to larger studies with reduced control over what actually happens in the field.  The intervention was implemented on top of an existing program, with a minimal involvement on the part of the researcher team. Our results indicate that despite a number of implementation problems, which were in part present because we wanted to work with a model that could be reproduced at scale, the enhancement we evaluated had a sizeable effect on the children most in need. |
| Is the intervention still ongoing at ≥ 12 months? | The intervention lasted 15 months approximately. However, the FAMI program is still ongoing. |
| Description of any long-term subsidies/incentives and plans for continuity or phase-out, and their effects on adoption/sustained use |  |
| Reference:  Attanasio, O., Baker-Henningham, H., Bernal, R., Meghir, C., Pineda, D., & Rubio-Codina, M. (2022). Early Stimulation and Nutrition: The Impacts of a Scalable Intervention. Journal of the European Economic Association, 20(4), 1395–1432. https://doi.org/10.1093/jeea/jvac005 | |

5. **Key elements linked to ECD intervention in Colombia from a perspective of the RE-AIM**

| **RE-AIM Element** | **ECD intervention in India: *Parenting intervention*** |
| --- | --- |
| **REACH** | |
| Description of target population (geographic coverage, numbers targeted, demographic characteristics) | Mothers with children aged 7 to 16 months. |
| Setting characteristics (urban vs. rural, etc.) | The intervention was implemented in three districts Cuttack, Salepur, and Bolangir |
| What percent of potentially eligible participants a) were excluded, b) took part and c) how representative were they? |  |
| Characteristics of participants reached compared to non-participants or to target population | Children were identified through pre-baseline household censuses and were deemed eligible if they were singletons, aged 7 to 16 months by the beginning of the intervention, and had no obvious disability. This age range was chosen because pregnant women often go away for their deliveries and most return home by 7 months. |
| Other factors that affect the reach of the program, including policy context, program budget constraints, conflict, and cost. |  |
| **EFFECTIVENESS** | |
| What were the goals of the program? | Multicomponent home-visiting intervention to improve ECD |
| What were the successes achieved toward stated goals of the program? | Over 2 years, home visiting had 0.324 SD impact on cognition (95% confidence interval [CI]: 0.152 to 0.496; stepdown P = .001) and a 0.239 SD on language (95% CI: 0.072 to 0.407; stepdown P = .009).  Group sessions had a 0.281 SD impact on cognition (95% CI: 0.100 to 0.463; stepdown P = .007) and a 0.302 SD on language (95% CI: 0.136 to 0.468; stepdown P = .001).  Differences in average impacts between home visiting and group sessions over 2 years were not significant.  For both cognition and language, there was no significant difference between the impact at midline and at endline for either home visiting or group sessions. Neither treatment arm affected motor skills.  The nutritional education arm had no significant impact on any of the Bayley-III outcomes. There were no significant differences among study arms in weight-for-age or height-for-age z scores |
| What impact did the intervention have on all participants who began the program? | Significantly improved child development among treated families. |
| What was the effect on process intermediate, and primary outcomes? |  |
| Possible negative or unintended consequences of the intervention on quality of life and related factors |  |
| Economic impact |  |
| **ADOPTION** |  |
| What level was the program/intervention targeting (e.g., individual, community, policy)? How representative were the participating settings? | Community level. |
| Description of regulations and legislation and how these affected adoption and continued use. |  |
| Other factors important to adoption |  |
| **IMPLEMENTATION** |  |
| Description of implementation strategy including underlying theory, if any, and how it may be integrated with any other interventions | Four study groups. All interventions were conducted weekly for 24 months. Home visits lasted ∼60 minutes, group meetings lasted 90 minutes, and nutritional education visits 40 minutes.  *Nutrition education arm* was delivered individually and was focused on improving the quality of children’s diets and basic hygienic practices in households through games, stories, and cooking demonstrations.  *Home visiting and group sessions* were both focused on psychosocial stimulation and included some nutritional education content. Stimulation was based on the Reach-Up and Learn home visiting program, which has a structured home visiting curriculum, previously adapted to the local.  Facilitators showed mothers how to play and interact with and respond to their children in ways likely to promote development.  They demonstrated play activities and encouraged mothers to participate by using toys made from locally available materials and purpose-designed books. Mothers were given the play materials to use at home and then exchanged weekly. |
| Cost of intervention (time or money) from the implementer perspective | Group sessions cost $38 per child per year and were as effective on average as home visiting, which cost $135, implying an increase by a factor of 3.5 in the returns to investment with group sessions, offering a more scalable model. |
| Consistency of implementation across staff/time/settings/subgroups (not about differential outcomes, but process) | The study strengths are its rigorous design, use of well-established developmental tests with midpoint evaluations, and use of a feasible delivery strategy, conducted by a national nongovernmental organization with readily available staff. |
| Adaptations made to intervention during program/project roll out (i.e., was the intervention delivered as intended?) | No important changes took place during implementation |
| Other factors important to implementation, including policy and regulatory environment. | Non-attendance at sessions: temporary migration for the home visiting and nutritional education arms and mothers being too busy for the group sessions arm.  A disadvantage of group sessions compared with home visiting was that more mothers refused to enroll, dropped out, or attended poorly. |
| **MAINTENANCE** |  |
| To what extent were different intervention components continued or institutionalized? How was the original program modified? | The intervention did not continue. |
| Availability/accessibility of intervention over time, and importance of these factors to adoption and sustained use |  |
| Is the intervention still ongoing at ≥ 12 months? | No, the intervention did not continue. |
| Description of any long-term subsidies/incentives and plans for continuity or phase-out, and their effects on adoption/sustained use |  |
| Reference:  Grantham-McGregor S, Adya A, Attanasio O, Augsburg B, Behrman J, Caeyers B, Day M, Jervis P, Kochar R, Makkar P, Meghir C, Phimister A, Rubio-Codina M, Vats K. Group Sessions or Home Visits for Early Childhood Development in India: A Cluster RCT. Pediatrics. 2020 Dec;146(6):e2020002725. doi: 10.1542/peds.2020-002725. Epub 2020 Nov 4. PMID: 33148771; PMCID: PMC7786825. | |
