# Supplementary material for: Strengthening Early Childhood Development Programs and Interventions in Low‐ and Middle‐Income Countries: Systematic Review of Interventions
Source: Matern Child Nutr. 2026 Sep 1;22(3):e70244. doi: 10.1111/mcn.70244 (PMC13531360; doi:10.1111/mcn.70244)
Supplement: Supplementary file 2 [file MCN-22-e70244-s002.docx]

**Supplementary Table 2.** Excluded studies in the systematic review

| **Reasons for exclusion** | **References** |
| --- | --- |
| Study design (protocol trail, multi-country) | (Jordan, B., et al., 2014; Cuartas, J., et al., 2023) |
| Does not report outcomes of interest | (Smith, J. A., et al., 2018; Healy, M. R., et al., 2024; Domek, G. J., et al., 2017; Kawser, U., et al., 2024; Ali, N. B., et al., 2024; Dusabe, C., et al., 2023; Luoto, J. E., et al., 2021; Bernal, R., et al., 2019) |
| Target population (only mothers, preterm infants, children over 5 years) | (Alves, C. R., et al., 2022, Yousafzai, A. K., et al., 201). |
| Repeated sample | (Baek, Y., et al., 2023; Luoto, J. E., et al., 2021) |
| High-income country | (Milman, H. M., et al., 2018) |

Ali, N. B., Yousafzai, A. K., Siyal, S., Bhamani, S., & Sudfeld, C. R. (2024). Effect of a Center-Based Early Childhood Care and Education Program on Child Nutritional Status: A Secondary Analysis of a Stepped-Wedge Cluster Randomized Controlled Trial in Rural Sindh, Pakistan. *J Nutr,* 154(2), 755-764. <https://doi.org/10.1016/j.tjnut.2023.12.008>

Alves, C. R., Gaspardo, C. M., Altafim, E. R., & Linhares, M. B. (2022). Effectiveness of a longitudinal psychosocial intervention to strengthen mother–child interactions: The role of biological and contextual moderators. *Children and Youth Services Review*, 2022, 13, 10633.

Baek, Y., Ademi, Z., Tran, T., Owen, A., Nguyen, T., Luchters, S., Hipgraves, D. V., Hanieh, S., Tran, T., Tran, H., Biggs, B., & Fisher, J. (2023). Considering equity and cost-effectiveness in assessing a parenting intervention to promote early childhood development in rural Vietnam. *Health Policy Plan,* 38(8), 916-925. hhtps://doi.org/doi:10.1093/heapol/czad057

Bernal, R., & Ramírez, S. M. (2019). Improving the quality of early childhood care at scale: The effects of “From Zero to Forever”. *World Development*, 118, 91-105. https://doi.org/ 10.1016/j.worlddev.2019.02.012

Cuartas, J., McCoy, D., Sánchez, J., Behrman, J., Cappa, C., Donati, G., Heymann, J., Lu, C., Raikes, A., Rao, N., Richter, L., Stein, A., & Yoshikawa, H. (2023). Family play, reading, and other stimulation and early childhood development in five low-and-middle-income countries. *Dev Sci,* 26(6), e13404. https://doi.org/10.1111/desc.13404.

Domek, G. J., Cunningham, M., Jimenez-Zambrano, A., Dunn, D., Abdel-Maksoud, M., Bronsert, M., Luna-Asturias, C., & Berman, S. (2017). Designing and Implementing an Early Childhood Health and Development Program in Rural, Southwest Guatemala: Lessons Learned and Future Directions. *Adv Pediatr,* 64(1), 381-401. https://doi.org/10.1016/j.yapd.2017.04.003

Dusabe, C., Abimpaye, M., Kabarungi, N., & Uwamahoro, M.D. (2023). Monitoring, evaluation and accountability evidence use for design, adaptation, and scale-up of an early childhood development program in Rwanda. *Front Public Health,* 11, 1165353. https://doi.org/10.3389/fpubh.2023.1165353.

Healy, M. R., Viegas da Silva, E., Lundborg, A. R., Hartwig, F. P., Munhoz, T. N., Arteche, A. X., Ramchandani, P. G., & Murray, J. (2024). Towards a better understanding of real-world home-visiting programs: a large-scale effectiveness study of parenting mechanisms in Brazil. *BMJ Glob Health,* 9(2), e013787. https://doi.org/10.1136/bmjgh-2023-013787.

Jordan, B., Tseng, Y. P., Coombs, N., Kennedy, A., & Borland, J.. (2014). Improving lifetime trajectories for vulnerable young children and families living with significant stress and social disadvantage: the early years education program randomised controlled trial. *BMC Public Health,* 14, 965. https://doi.org/10.1186/1471-2458-14-965.

Kawser, U., Driscoll, J., & Huque, A. U. (2024). Parent Education in Bangladeshi Families with Young Children of Diverse Abilities: Implementation of the Learning Through Play Calendar. *International Journal of Systemic Therapy,* 35(3), 310–326. <https://doi.org/10.1080/2692398X.2024.2325757>

Luoto, J. E., Lopez Garcia, I., Aboud, F. E., Singla, D. R., Fernald, L. C., H., Pitchik, H. O., Saya, U. Y., Otieno, R., & Alu, E. (2021). Group-based parenting interventions to promote child development in rural Kenya: a multi-arm, cluster-randomised community effectiveness trial. *Lancet Glob Health.* 9(3):e309-e319. https://doi.org/doi: 10.1016/S2214-109X(20)30469-1

Luoto, J. E., Lopez Garcia, I., Aboud, F. E., Singla, D. R., Zhu, R., Otieno, R., & Alu, E. (2021). An Implementation Evaluation of A Group-Based Parenting Intervention to Promote Early Childhood Development in Rural Kenya. *Front Public Health,* 9, 653106. https://doi/10.3389/fpubh.2021.653106.

Milman, H. M., Castillo, C. A., Sansotta, A. T., Delpiano, P. V., & Murray, J. (2018). Scaling up an early childhood development programme through a national multisectoral approach to social protection: lessons from Chile Crece Contigo. *BMJ*, 363:k4513. https://doi.org/doi:10.1136/bmj.k4513

Smith, J.A., Baker-Henningham, H., Brentani, A., Mugweni, R., & Walker, S. P. (2018). Implementation of Reach Up early childhood parenting program: acceptability, appropriateness, and feasibility in Brazil and Zimbabwe. *Ann N Y Acad Sci,* 1419(1), 120-140. https://doi.org/10.1111/nyas.13678

Yousafzai, A. K., Rasheed, M. A., Rizvi, A., Shaheen, F., Ponguta, L. A., & Reyes, C. R. (2018). Effectiveness of a youth-led early childhood care and education programme in rural Pakistan: A cluster-randomised controlled trial. *PLoS One,* 13(12), e0208335. https://doi.org/10.1371/journal.pone.0208335.
