# Supplementary material for: Strengthening Early Childhood Development Programs and Interventions in Low‐ and Middle‐Income Countries: Systematic Review of Interventions
Source: Matern Child Nutr. 2026 Sep 1;22(3):e70244. doi: 10.1111/mcn.70244 (PMC13531360; doi:10.1111/mcn.70244)
Supplement: Supplementary file 3 [file MCN-22-e70244-s003.docx]

**Supplementary Table 1.** Databases and individualized search strings.

| **Database** | **Search string** |
| --- | --- |
| PubMed Central | *Search string 1*  (nurturing care AND early childhood development) AND (intervention programs) AND (low-middle income countries OR developing countries) AND (benefit cost-ratio)  *Results: 5* |
|  | *Search string 2*  (nurturing care interventions) AND (early child* development) AND (LMIC) AND (cost effectiveness)  *Results: 114* |
|  | *Search string 3*  (family OR parenting OR home-visits) AND (early childhood development) AND (low-middle income countries OR developing countries) AND (cost effectiveness)  *Results: 21,451 *Note: The first 5 result pages were reviewed and 100 records were retrieved.* |
| Web Of Science | *Search string 1*  (((ALL=(nurturing care)) AND ALL=(early childhood development)) AND ALL=(intervention programs)) AND ALL=(low-middle income countries or developing countries)  *Results: 16* |
|  | *Search string 2*  (((ALL=(nurturing care interventions)) AND ALL=(early child* development )) AND ALL=(LMIC))  *Results: 4* |
|  | *Search string 3*  (((ALL=(family or parenting)) AND ALL=(early childhood development)) AND ALL=(low-middle income countries OR developing countries)) AND ALL=(cost effectiveness)  *Results: 14* |
| Scopus | *Search string 1*  ( nurturing care ) AND ( early childhood development ) AND ( intervention programs ) AND ( low-middle income countries OR developing countries ) AND PUBYEAR > 2013 AND PUBYEAR < 2025 AND ( LIMIT-TO ( DOCTYPE , "ar" ) OR LIMIT-TO ( DOCTYPE , "re" ) ) AND ( LIMIT-TO ( LANGUAGE , "English" ) OR LIMIT-TO ( LANGUAGE , "Portuguese" ) )  *Results: 80* |
|  | *Search string 2*  ( nurturing care interventions ) AND ( early child* development ) AND ( LMIC ) AND ( cost effectiveness ) AND PUBYEAR > 2016 AND PUBYEAR < 2025 AND ( LIMIT-TO ( DOCTYPE , "ar" ) OR LIMIT-TO ( DOCTYPE , "re" ) ) AND ( LIMIT-TO ( LANGUAGE , "English" ) )  *Results: 14* |
|  | *Search string 3*  ( family OR parenting OR home-visits ) AND ( early childhood development ) AND ( low-middle income countries OR developing countries ) AND ( cost effectiveness ) AND PUBYEAR > 2013 AND PUBYEAR < 2025 AND ( LIMIT-TO ( DOCTYPE , "ar" ) OR LIMIT-TO ( DOCTYPE , "re" ) ) AND ( LIMIT-TO ( LANGUAGE , "English" ) )  *Results: 204* |
| ScienceDirect | *Search string 1*  (nurturing care) AND (early childhood development) AND (intervention programs) AND (low-middle income countries OR developing countries) AND (benefit cost-ratio)Year:2014-2024  *Results: 74* |
|  | *Search string 2*  (nurturing care interventions) AND (early child development) AND (LMIC) AND (cost effectiveness)Year:2014-2024  *Results: 75* |
|  | *Search string 3*  (family OR parenting OR home-visits) and early childhood development and (low-middle income countries or developing countries) AND (cost effectiveness)Year:2014-2024  *Results: 1128*  **Note: The first 5 result pages were reviewed, and 75 studies were retrieved.* |
| MEDLINE | *Search string 1*  nurturing care AND early childhood development AND intervention programs  *Results: 5* |
|  | *Search string 2*  nurturing care interventions AND early child development AND (lmic OR low income countries or middle income countries OR developing countries)  *Results: 1* |
|  | *Search string 3*  (family OR parenting OR home-visits) and early childhood development and (low-middle income countries or developing countries) AND (cost effectiveness)  *Results: 2* |
