# Supplementary material for: Strengthening Early Childhood Development Programs and Interventions in Low‐ and Middle‐Income Countries: Systematic Review of Interventions
Source: Matern Child Nutr. 2026 Sep 1;22(3):e70244. doi: 10.1111/mcn.70244 (PMC13531360; doi:10.1111/mcn.70244)
Supplement: Supplementary file 4 [file MCN-22-e70244-s004.docx]

**Supplementary Material 3.** Main characteristics of the included studies' programs and interventions (n= 34).

| **Authors** | **Year** | **Country** | **Design** | **Intervention / Program** | **Domains** | **Target population** | **Outcomes** | **Results** | **Quality assessment** | |
| --- | --- | --- | --- | --- | --- | --- | --- | --- | --- | --- |
|  |  |  |  |  |  |  |  |  | **Internal** | **External** |
| **Region of the Americas** | | | | | | | | | | |
| Weisleder et al. | 2018 | Brazil | CRCT | Intervention | Opportunities for early learning Responsive caregiving | Caregiver–child dyads  Low-income children ages 2 to 4 years | Child development (Cognitive stimulation assessed by StimQ, a structured interview; receptive vocabulary by Teste de Vocabulário  por Imagens Peabody (TVIP); expressive vocabulary by the Teste Infantil de Nomeação; working memory by Teste Infantil  de Memória de Trabalho (TIMT); IQ by Brazilian version of the Snijders-Oomen nonverbal  intelligence test (SON-R)) | After adjustment, children in the intervention group (IG) had higher receptive vocabulary scores (mean difference [MD] = 2.54, 95% CI: 1.10-3.97, P = 0.001), working memory scores (MD= 2.20, 95% CI: 1.0-3.41, P < 0.001), and IQ scores (MD= 5.58, 95% CI: 2.98-8.17, P < 0.001) than children in the control group (CG). There were no significant group differences in expressive vocabulary (P = 0.24) or phonological short-term memory (P = 0.29) and children’s social-emotional competence (P = 0.56) or externalizing behaviors (P =0.24).  Differences between IG and CG: cognitive stimulation (MD StimQ Total= 2.77, 95% CI, 0.21-5.34, P = 0.03), especially in the context of reading activities (MD StimQ READ= 1.84, 95% CI: 0.76-2.92, P < 0.001), and observed interactive reading (MD ACIRI = 2.80, 95% CI: 1.40-4.19, P < 0.001). | Moderate | Moderate |
| González-Fernández et al. | 2020 | Peru | Quasi-experimental | Intervention | Good health  Adequate nutrition  Responsive caregiving  Opportunities for early learning Security and safety | Children 0-3 years old | Child development (Integrated Management of Childhood  Illness (IMCI))  Food Insecurity  Malnutrition | The level of food insecurity did not differ between control communities (CC) and intervention communities (IC) at 8 or 12 months.  The diversity of foods consumed by children increased more than 2-fold over the study in both CC and IC.  The number of doctor visits and episodes of diarrhea and respiratory infections in the IC declined at 8 and 12 months (p = 0.0001).  Increase in hemoglobin concentrations and decreased percentages of anemia in both IC and CC throughout evaluations.  In general, children from CC and IC were within the normal weight range, with a very low prevalence of WAZ. At 8 and 12 months, the LAZ was lower in IC children than in CC children.  At 8 months, after the intervention with gardens and nutrition, the proportion classified as “developmental delay alert” was lower in the IC (8.9%) compared with the CC (20%). There was no significant difference in the proportion of any developmental groups within communities over time (p= 0.056).  The subjective caregiver evaluation showed that all IC parents felt they had improved their skills and qualities as caregivers.  Receiving the intervention was associated with a 60% decreased risk of language delay. | Moderate | Moderate |
| Buccini et al.^1^ | 2021 | Brazil | Qualitative | Program | Good health  Responsive caregiving  Security and safety  Adequate nutrition  Opportunities for early learning | Pregnant women and children 0-11 months | Child development | N.A. | Adequate | Adequate |
| Santos et al. | 2022 | Brazil | RCT | Program | Good health  Responsive caregiving  Security and safety  Adequate nutrition  Opportunities for early learning | Pregnant women and children aged 0-11 months | Child development (Integral child development: questionnaires to the children’s mothers adapted from the UNICEF Multiple Indicator Cluster Survey (MICS) and the Pelotas Birth  Cohort Studies; Ages and Stages Questionnaire (ASQ3). | 1-year follow-up: 31.3% of the children in IG had been visited the previous month, and 24.1% of the mothers recalled any advice the visitors provided.  The mean ASQ-3 score was 2.4 points higher in the IG than in the comparison group (CG) but was not statistically significant (p= 0.145).  3-year follow-up: The mean stimulation-interactions scores were very similar for the IG and CG for both age ranges studied. The mean scores were about 80% of the scale's maximum values. The responsive caregiving scores derived from the videos recorded for a subsample were relatively low (2.1 out of a maximum of 5.0) in both groups.  There were no differences in the ITT and LATE analyses for responsive caregiving, disciplinary methods, or psychological attributes. | Moderate | Moderate |
| Viegas da Silva | 2022 | Brazil | Cohort | Program | Good health  Adequate nutrition  Responsive caregiving  Opportunities for early learning | Families enrolled at any time between pregnancy and child age 4 years | Child development (Battelle’s Developmental Inventory  (BDI) -five neurodevelopment domains: personal-social, adaptive, motor, communication and cognitive-) | Comparing Primeira Infância Melhor program (PIM) children (enrolled at any time) with matched controls, there was no strong statistical evidence that PIM affected child development measured at age 4.  PIM starting during pregnancy was associated with 0.19 SD (95% CI -0.02-0.40), higher development scores at age 4, and 60% lower prevalence (prevalence ratio=0.40; 95% CI 0.18-0.89) of having a low development score (below the 10th percentile).  In contrast, there was no strong statistical evidence of association with child outcomes for PIM starting after birth. | Moderate | Adequate |
| Attanasio et al. | 2022 | Colombia | CRCT | Intervention | Good health  Adequate nutrition  Responsive caregiving Opportunities for early learning | Children aged 0-24 months | Height-for-age Z-scores  Socio-emotional development (Bayley-III)  Food insecurity | The effect of the program on the Bayley-III factor was 0.163 SD.  The program has no significant average impact on socio-emotional development or HAZ.  The fraction of children whose height-for-age was below -1 SD decreased by 6.8 percentage points or 0.15 SD, while the number of children with normal height-for-age increased by a similar fraction (7.6 percentage points) (p <0.05).  No statistically significant program effects on maternal knowledge about child development, maternal self-efficacy, or food insecurity. | Moderate | Moderate |
| Nuño et al. | 2022 | Peru | CRCT | Intervention | Good health  Adequate nutrition  Security and safety | Children aged 0-36 months | Child development (Age standardised mean scores of socio-emotional, fine and gross motor, communication, and cognitive skills and an overall performance:  nationally validated Peruvian Infant Development Scale (ESDI) and the Spanish version of the internationally validated Bayley Scales of Infant and Toddler Development (BSID)) | Children from the ECD combined arm scored better in all Peruvian Infant Development Scale (ESDI) domains (difference range: 9–25%). The highest differences were observed in the cognitive domain (OR: 1.9; 95% CI: 1.1–3.5) and overall performance (OR: 2.8; 95% CI: 1.6–4.9).  Any change in anthropometric status between arms at end-of-study was observed. | Moderate | Moderate |
| Waechter et al. | 2022 | Grenada and West Indies | RCT | Intervention | Good health  Opportunities for early learning  Safety and security | Children 0-2 years old | Neurodevelopmental measure (INTERGROWTH-21st Neurodevelopment Assessment (INTER-NDA))  Corporal punishment | The mixed model analysis results showed that children whose caregivers received the conscious discipline (CD) intervention scored significantly higher across the INTER-NDA domains of cognition, fine motor, gross motor, and language than those allocated to the waitlist control group (CG).  The effect sizes for the CD group ranged from small (0.216 for cognition) to medium (0.524 for fine motor). The CD intervention accounted for 0.524 (medium effect) of the performance in the fine motor domain.  There were no significant differences in corporal punishment beliefs, attitudes, and behaviors between the CD group and the waitlist CG. | Moderate | Moderate |
| **Africa Region** | | | | | | | | | | |
| Saori et al. | 2019 | Rwanda | Quasi-experimental | Intervention | Responsive caregiving  Opportunities for early learning | Children 3-6 years old | Child development -Motor, literacy, numeracy, social-emotional development (International Development and Early Learning Assessment (IDELA)) | The program had the largest impact on the emergent literacy domain, followed by the emergent numeracy domain.  On average, children who scored high on baseline numeracy, literacy, socio-emotional, or motor scored higher on the endline IDELA than those who did not. Older children scored higher than younger children. Higher mothers’ and fathers’ education levels also predicted higher IDELA scores compared to children with parents whose educational attainment was lower. The equity analysis found that the program seems to have a stronger impact on children with literate fathers. | Low | Low |
| López-García et al. | 2021 | Kenya | RCT | Intervention | Good health  Opportunities for early learning | Mothers or female primary caregivers aged 15 years or older with children aged 6–24 months | Child development -cognitive and receptive language development- (Bayley Scales of Infant Development, Third Edition) | The group-only and the mixed-delivery models significantly improved child cognitive and socioemotional development relative to the control group, and the group-only model also showed statistically significant improvements in child receptive language.  The intention-to-treat (ITT) effect sizes of improvements in the group-only arm were 0.52 standard deviation (SD; p = 0.001) in cognition, 0.42 SD (p = 0.017) in receptive language, and 0.23 SD (p = 0.024) in socio-emotional development.  In the mixed-delivery arm, the ITT effect sizes for cognition were 0.34 SD (p = 0.021), 0.20 SD (p = 0.17) for receptive language, and 0.22 SD (p = 0.011) for socio-emotional development. | Moderate | Moderate |
| Jensen et al. | 2021 | Rwanda | CRCT | Intervention | Good health  Responsive caregiving  Security and safety | Families with children aged 6–36 months | Child development  -children’s motor, language, cognitive and social development-  (Ages and Stages Questionnaire (ASQ-3) and Malawi Development Assessment Tool (MDAT)  Children anthropometric growth | Intervention families improved more across the domains of gross motor (β=0.294, 95% CI: 0.118-0.470), communication (β=0.139 95% CI 0.009-0.268), problem-solving (β=0.159, 95% CI 0.035-0.282) and personal-social development (β=0.148, 95% CI 0.023-0.273).  There are no significant group differences in change in fine motor development.  Among families with a father in the household, father engagement increased more in families receiving Sugira Muryango (SM) compared with usual care (UC) (OR=1.592, 95% CI 1.069-2.368). Families receiving SM also showed a greater decrease in the use of harsh discipline (IRR=0.741, 95% CI 0.657-0.835).  Among those in dual-caregiver households, female caregivers receiving SM showed a greater decrease in intimate partner violence (IRR=0.616, 95% CI 0.458-0.828).  At 12-month follow-up, no significant intervention effects were observed on HAZ, WAZ, WHZ, or MUAC (all 95% CIs crossed zero). | Moderate | Moderate |
| Russell et al. | 2022 | Tanzania | Cross-sectional | Program | Responsive caregiving  Adequate nutrition  Opportunities for early learning | Children aged 18-29 months and their caregivers | Child cognitive development (Caregiver  Reported Early Developmental Index (CREDI)) | An increased number of early stimulating activities was associated with improved child development outcomes across all CREDI domains (cognitive, language, social-emotional, and motor) (adjusted p-values: 0.002 to 0.017).  Each additional activity any caregiver does with their child was associated with a 0.036 point increase in overall CREDI score (95% CI = (0.014, 0.058), p = 0.002).  The strongest associations were seen in the motor and language domains, where one additional activity was associated with a 0.039 point increase (95% CI = (0.011, 0.066), p = 0.007) in motor development and 0.039 point increase (95% CI = (0.007, 0.053), p = 0.017) in language development CREDI scores in the adjusted model. | Moderate | Moderate |
| Desmond et al. | 2023 | Rwanda | CRCT | Intervention | Good health  Responsive caregiving  Security and safety | Families with children aged 6–36 months | Child development  -Cognitive development-  (Ages and Stages Questionnaire (ASQ-3)) | -Standardized impact on cognitive development: 0.11 SD. -Number of home visits/group sessions (intended): 12/0 -The improvement in cognitive development in SDs. per session is 0.009 -Sugira Muryango involves far fewer sessions than similar interventions. | Moderate | Moderate |
| Ahmed et al. | 2023 | Uganda | CRCT | Intervention | Good health  Adequate nutrition  Opportunities for early learning | Mother-child dyads (children under 2 years old, recruited when at 6–8 months old) | Child development  -Cognitive development-  (Bayley Scales of Infant and Toddler Development-III) | The educational intervention was effective in improving cognitive development among small children. Specifically, when the children were aged 20–24 months, children in the intervention group had, on average, 15.30 units higher cognitive composite scores than those assigned to the control group (114.67 versus 99.37; p = 0.000). | Adequate | Moderate |
| Skeen et al. | 2023 | Zambia and Tanzania | RCT | Intervention | Good health  Opportunities for early learning  Security and safety | Caregiver–child dyads (children between 9 and 32 months of age at the time of recruitment) | Socio-emotional development (CREDI social–emotional long form)  Caregiver-reported responsive caregiving  Child language (Caregiver  Reported Early Developmental Index (CREDI)) | Post-intervention caregivers in the intervention group (IG) were significantly more likely to be engaging in responsive caregiving practices (OR = 2.55, 95% CI: 1.15–5.66, p = 0.02) than caregivers in the control group (CG).  No significant improvements in child social and emotional development or child language were observed in the IG compared to children in the CG.  For Zambia, no child development or parenting outcomes were statistically significant; however, a positive trend (p <0.1) was detected for telling the child stories (β=0.65, 95% CI: −0.03–1.32, p=0.06) and talking to the child during meals (β=0.65, 95% CI: −0.07–1.37, p=0.08).  For Tanzania, caregivers in the IG were significantly more likely to engage in responsive caregiving practices (OR=4.49, 95% CI: 1.32–15.28, p=0.02) and spend time telling their child stories (β=0.80, p=0.01).  Caregivers reported significant increases in observed peer problems in IG children compared to CG (β=0.46, p=0.04). | Moderate | Moderate |
| Leighton et al. | 2023 | Tanzania | Quasi-experimental | Intervention | Good health  Responsive caregiving  Opportunities for early learning | Children aged 4-12 months | Child development  -motor, cognitive, language and socio-emotional development-  (Caregiver  Reported Early Developmental Index (CREDI)) | After two years of implementation, the intervention (IG) improved the development of 3-year-olds by 0.203 standard deviations (SD) compared with the reference population, while the control group (CG) fell slightly behind the reference population, scoring 0.089 SD below the reference.  The overall treatment effect, combining both treatment arms, is 0.287 and highly significant; this indicates that children in the IG had 28.7% of a SD higher child development score compared with similar children in the CG.  The treatment effect was not statistically significant (0.270 vs. 0.306) across the two treatment arms. | Moderate | Moderate |
| **Western Pacific Region** | | | | | | | | | | |
| Pisani et al. | 2016 | Cambodia | Pre-post (before-and-after) | Intervention | Opportunities for early learning | Children under 6 years old | Child development  -Motor, literacy, numeracy, social-emotional development-  (International Development and Early Learning Assessment (IDELA)) | There were no significant differences between boys’ and girls’ socioemotional development.  The children had fairly equal skills in all the subareas assessed and, on average, correctly completed 43% of items. In the area of emergent literacy, children had the strongest skills in print awareness and oral comprehension and the weakest in letter identification.  There were no gender differences in IDELA scores, but age was significantly correlated, with older children scoring significantly higher than younger children. | Low | Low |
| Zhou et al. | 2019 | China | Quasi-experimental | Intervention | Good health  Adequate nutrition  Responsive caregiving  Opportunities for early learning Security and safety | Children aged 0 to 35 months and their primary caregivers | Neurodevelopmental delay  (Nationally validated Chinese version of the Ages & Stages  Questionnaire (ASQ), third edition) | Prevalence of overall suspected neurodevelopmental delay (SDD) decreased from 37% at baseline to 19% post-intervention [-18%,95% CI: 0.69 (0.54-0.89), P= 0.005] (versus 10% in comparison village) absolute reduction.  Intervention villages had significantly lower odds of overall SDD (difference-in-differences (DID) aOR= 0.69, 95% CI: 0.54–0.89) than comparison villages.  Reductions across specific developmental domains, including communication, gross motor, and fine motor skills (-10%, 95% CI: 0.69 (0.48-0.98); -9%, 95% CI: 0.65 (0.44-0.97); -13%, 95% CI: 0.40 (0.27-0.59), respectively).  Higher ECD center intervention exposure and higher clinic exposure improved the Ages & Stages Questionnaire (ASQ) score by increasing the frequency of cognitive stimulation and positive discipline behavior at home, as well as the length-for-age Z score (LAZ) and hemoglobin concentration. | Moderate | Adequate |
| Luo et al. | 2020 | China | CRCT | Intervention | Good health  Adequate nutrition  Responsive caregiving Opportunities for early learning | Children 6–18 month-old and their main caregiver | Child development  -Cognition, language, motor, and social-  emotional skill development-  (Bayley Scales of Infant and Toddler Development-III)  Health (morbidity, nutrition, and growth)  Measures of parenting practices | The integrated parenting intervention significantly improved the summary index of skill development (effect size [ES] 0.17 SD, 95% CI 0.02 SD-0.33 SD). Intention-to-treat (ITT) effects showed that the intervention improved cognitive development by 0.24 standard deviations (SD) [95% CI 0.04 SD-0.44 SD].  No effect on (receptive or expressive) language, (gross or fine) motor, or social-emotional development.  The intervention improved overall caregiver-reported child health (ES 0.29 SD, CI 95% 0.13 SD-0.46 SD).  The intervention reduced 8.1 [95% CI 3.8–12.4] percentage points in the risk that the child had an episode of diarrhea during the two-week period prior to the survey.  The intervention did not find a significant effect on child nutrition or growth outcomes. | Adequate | Moderate |
| Shi et al. | 2020 | China | CRCT | Intervention | Good health  Responsive caregiving Opportunities for early learning | Infants aged 1-2 months and their caregivers | Child development  -Communication, gross motor skills, fine motor skills, problem-solving, and personal-social development-  (Chinese version of the Ages & Stages Questionnaires third edition (ASQ-C)) | Children in the intervention group: significantly higher scores in communication (aMD= 0.26; 95% CI 0.03, 0.51), fine motor skills (aMD= 0.19; 95% CI 0.01, 0.37), and overall development (aMD= 0.25; 95% CI 0.10, 0.41) as measured by the ASQ at 14 months of age compared to the control group.  Results indicate that the parenting intervention integrated with primary health care effectively enhanced early childhood development outcomes. | Adequate | Adequate |
| Sylvia et al. | 2022 | China | CRCT | Intervention | Good health  Responsive caregiving Opportunities for early learning | Children age 6–24 months and their caregivers | Child development  -Cognitive, language, motor, and social-emotional skills-  (Bayley Scales of Infant Development-III) | The center-based parenting intervention did not significantly improve the development outcomes (skills) of children in the program.  The intervention increased caregiver investments in children, including material investments in toys and picture books, increased time investments in stimulating parenting activities, and improved parenting skills.  The center-based parenting intervention was significantly less effective than the home-based intervention, producing significantly smaller average impacts on child skill development and caregiver investments.  Whereas parents of children with low baseline cognitive skills tended to select out of the center-based parenting program, the home-visiting parenting program effectively provided parental training to children with both low and high baseline cognitive skills. | Moderate | Moderate |
| Baek et al. | 2023 | Vietnam | CRCT | Intervention | Good health, responsive caregiving, security, and safety | Pregnant women/mothers and children under 2 years old | Child cognitive development (Bayley Scales of Infant Development-III) | There was a significant improvement in the child’s cognitive, language, and motor development scores. The mean difference score between control vs intervention group was 4.0 (95% CI 2.5 to 5.5) for cognitive development, 2.5 (95% CI 0.5 to 4.4) for language development, and 2.7 (95% CI 1.2 to 4.2) for motor development. The social–emotional development score showed no significant difference between groups: the mean difference was 2.3 (–1.5 to 6.1). | Moderate | Adequate |
| Li et al. | 2024 | China | Quasi-experimental | Intervention | Good health  Adequate nutrition  Responsive caregiving  Opportunities for early learning Security and safety | Left-behind children aged 0–35 months and their primary caregivers | Child development  -Communication, gross motor, fine motor, problem solving, and personal social-  (Chinese version of Age and Stages Questionnaires) | Children's early development in the intervention group (IG) showed significant improvement compared with the control group (CG) (OR = 0.59, 95% CI: 0.36, 0.96).  No significant differences between the groups were observed in either the caregiver's early stimulation or family learning materials.  Migrant parents of children in the intervention group showed a higher proportion of expressing emotional support and praise for their children when communicating (OR = 1.69, 95% CI: 1.05, 2.72).  Children who received home visits more than once every two months had a lower level of suspected development delay than children in the CG (OR = 0.34, 95% CI: 0.18, 0.68), while no significant difference was shown between children in the low-frequency group and the CG (OR = 0.72, 95% CI: 0.46, 1.15).  The high frequency of home visits received was associated with improved outcomes above. | Moderate | Adequate |
| **Eastern Mediterranean Region** | | | | | | | | | | |
| Bliznashka, et al. | 2022 | Pakistan | CRCT | Intervention | Responsive caregiving  Adequate nutrition  Opportunities for early learning  Good health | Mother‐child pairs child: <2.5 months - 24 months of age | Child development  -Cognitive,  language, motor, and socio‐emotional-  (Bayley Scales of  Infant and Toddler Development-III)  Linear growth  Child diet | All interventions improved cognitive, language, and motor scores at 24 months (Responsive stimulation [RS], cognitive: β 0.328, language β 0,321, motor β 0,263; enhanced nutrition [EN], cognitive: β 0.111, language β 0.211, motor β 0.119; RS+EN cognitive: β 0.229, language β 0.285, motor β 0.119).  Dietary diversity score (DDS) and Mother‐Child Interaction (OMCI) improved at 12 months.  None of the interventions improved socio‐emotional scores or LAZ at 24 months.  Higher OMCI at 12 months predicted higher development scores in all four domains at 24 months, whereas higher DDS at 12 months predicted higher LAZ, cognitive, and language scores at 24 months.  After controlling for child diet and mother-child interactions as mediators, the RS and RS + EN interventions directly predicted lower LAZ at 24 months.  The RS and RS + EN interventions improved cognitive, language, and motor scores at 24 months, whereas the EN intervention only improved language scores.  None of the interventions improved child growth. | Adequate | Adequate |
| Bemanalizadeh et al. | 2022 | Iran | RCT | Intervention | Good health  Opportunities for early learning  Responsive caregiving | Pregnant women aged 18–45 years in their third trimester and followed their children for 18 months | Child development  -Cognition, language, and motor-  (Bayley Scales of  Infant and Toddler Development-III) | The adjusted median/mean differences between intervention and control groups using median/linear regression were not significant for all Bayley Scales of Infant and Toddler Development-III (BSID-III) domains, except for median differences for cognitive score based on BSID-III (β (SE): − 4.98(2.31), p:0.032) and mean differences for anxiety/depression score based on Children Behavior Checklist (CBCL) (β (SE): − 2.54(1.27), p:0.046). | Moderate | Low |
| **South-East Asian Region** | | | | | | | | | | |
| Tofail et al. | 2018 | Bangladesh | CRCT | Intervention | Good health  Adequate nutrition  Security and safety | Pregnant women and children aged 6-24 months | Child development  -Communication, gross motor,  and personal social development-  (Extended Ages  and Stages Questionnaire (EASQ))  Gross motor milestones  Responsive parenting (Home  Measurement for Observation for the Environment (HOME)) | At year 2 follow-up, compared with the control group (CG), all intervention groups (IGs) except the water treatment group had higher scores on the communication and gross motor subscales, and all intervention groups had higher scores on the personal social subscale.  Compared with the CG, responsive parenting assessed by the HOME scales was improved at year 1 in the sanitation group, handwashing group, and combined water, sanitation, handwashing, and nutrition group, and at year 2 in the water treatment group, nutrition group, and combined water, sanitation, handwashing, and nutrition group. | Adequate | Adequate |
| Rosales et al. | 2019 | Armenia | Quasi-experimental | Intervention | Good health  Adequate nutrition  Responsive caregiving  Opportunities for early learning | Mothers and children aged 0 to 24 months | Child development  -Cognitive,  language, motor, and socio‐emotional-  (Bayley Scales of  Infant and Toddler Development-III)  Nutrition status  Parental childcare  Nutrition practices | The intervention demonstrated effectiveness in improving parental practices related to childcare and nutrition.  Parents in the intervention communities showed a 55% higher likelihood of providing minimum dietary diversity than control communities (aOR = 1.55, 95% CI: 1.10–2.19, P = 0.013).  There was a significant improvement in support for learning and disciplining practices among parents in the intervention group (aOR = 2.22, 95% CI 1.19–4.16, P = 0.012).  The intervention did not significantly affect children's nutritional status (Stunting aOR = 1.11, 95% CI 0.83–1.48, P = 0.501).  Children exposed to the integrated ECD intervention had better developmental outcomes in cognitive and language skills, especially in socioeconomically disadvantaged areas. | Moderate | Low |
| Grantham-McGregor, et al. | 2020 | India | CRCT | Intervention | Good health  Adequate nutrition  Responsive caregiving Opportunities for early learning | Children aged 7-16 months | Child development  -Cognitive, language, and motor development-  (Ages and Stages Questionnaire, third  edition (ASQ-3 and Bayley Scales of Infant and Toddler  Development-III))  Nutritional status | First year: The home visiting group improved significantly in cognition but not language, whereas the group sessions improved significantly in cognition and language.  Over 2 years, home visiting had a 0.324 SD impact on cognition (95% CI: 0.152-0.496; stepdown P = .001) and a 0.239 SD on language (95% CI: 0.072-0.407; stepdown P = .009).  Group sessions had a 0.281 SD impact on cognition (95% CI: 0.100-0.463; stepdown P = .007) and a 0.302 SD on language (95% CI: 0.136-0.468; stepdown P = .001). Differences in average impacts between home visiting and group sessions over 2 years were not significant.  For both cognition and language, there was no significant difference between the impact at the midline and the endline for either home visiting or group sessions. Neither treatment arm affected motor skills.  The nutritional education arm had no significant impact on any Bayley-III outcomes, and there were no significant differences among study arms in weight-for-age or height-for-age z scores. | Moderate | Adequate |
| Spier et al. | 2020 | Bangladesh | RCT | Intervention | Opportunities for early learning | Children 4 years old | Child development  -Motor, literacy, numeracy, social-emotional development-  (International Development and Early Learning Assessment (IDELA)) | The intervention had a lasting, positive intent-to-treat (ITT) impact of 0.23 standard deviations (SD) on children’s literacy, 0.30 SD on numeracy, and 0.34 SD on social and emotional development.  When compared to children’s natural progress when the intervention was not available in their communities, on average, after the mandatory preschool year (the difference in midline scores and endline scores for children in the control group), an additional year of preschool was found to increase literacy, numeracy, and social-emotional development scores by 18%, 26%, and 38%, respectively. | Moderate | Adequate |
| Hossain et al. | 2022 | Bangladesh | CRCT | Intervention | Good health  Adequate nutrition  Opportunities for early learning  Responsive caregiving | Rural poor women and their children aged 6-16 months at enrollment | Children's cognitive, language, and motor composite scores  (Bayley Scales of Infant and Toddler Development third edition)  BMI  Mothers' self-esteem | The intervention combining psychosocial stimulation (PS) and unconditional cash transfers (UCT) significantly improved cognitive and language development among children aged 6-16 months.  Children in the PS + UCT group showed a notable increase in cognitive scores (B= 2.96, p= 0.021, Effect size [ES]= 0.24) and language scores (B= 2.73, p = 0.022, ES= 0.21) compared to those receiving only UCT.  The PS + UCT group had even larger improvements than the standard care group, with cognitive scores (B= 3.73, p= 0.003, ES= 0.32) and language scores (B= 2.82, p= 0.016, ES= 0.24) significantly higher than those of the other groups.  The intervention positively impacted maternal self-esteem and home stimulation practices.  There was no apparent intervention effect on children's growth and mothers' BMI. | Adequate | Adequate |
| Kamiya and Nomura | 2023 | Lao PDR | Cross-sectional | Program | Responsive caregiving  Opportunities for early learning | Children aged 3-4 years old | Child development index  -Literacy and numeracy skills, readiness to learn, physical growth, and social-emotional  development-  (Early Childhood Development Index (ECDI) of MICS developed by UNICEF)  Home stimulation | Whether the child was enrolled in preschool, there was a statistically significant and positive effect on all four Early Childhood Development Index (ECDI).  The estimated OR for literacy-numeracy (OR = 4.754, p < 0.01) indicates that if the child attends preschool, the odds of a child being on track in literacy-numeracy increased by 4.754.  For learning (OR = 2.009, p < 0.01), physical growth (OR = 3.867, p < 0.01), and social-emotional development (OR = 1.188, p < 0.1) showed positive effects of preschool attendance on ECD.  Home stimulation by caregivers, statistically significant and positive effects were confirmed for literacy-numeracy (OR = 2.384, p < 0.01), learning (OR = 1.359, p < 0.05), and social-emotional (OR = 1.388, p < 0.01), but not for physical growth.  The estimated ORs showed that preschool attendance had larger effects than home stimulation in terms of literacy-numeracy, learning, and physical growth.  In contrast, a larger effect of home stimulation was confirmed for socioemotional development. | Moderate | Moderate |
| Gaidhane et al. | 2023 | India | CRCT | Intervention | Good health  Adequate nutrition  Responsive caregiving  Security and safety | Pregnant women and their children from birth to 2 years old | Child development  -Motor, language, personal-social, and emotional development-  (Developmental Milestones Checklist (DMC) and Profile  of Socio-Emotional Development (PSED))  -Quality mother interactions (Observation of the Mother–Child  Interaction (OMCI))  Nutrition status | The intervention group (IG) had a lower proportion of low-birth-weight newborns (20.86%) than the control group (CG) (26.67%), but this difference was not statistically significant.  The birth weight was significantly higher in the IG 2.71 (SD 0.44) than in the CG 2.61 (SD 0.45). Ninety-six percent of women started breastfeeding within 1 h of delivery, and the exclusive breastfeeding rate was 89.80%.  Statistically significant effect on weight for height (WHZ) in children at 24 months in the intervention group (−1.47 (1.13; −1.60 −1.34), p=0.033) was identified, but no for WAZ or HAZ in IG at 24 months, (−1.73 (1.01; −1.85 −1.62), p= 0.515);(1.37 (1.09; −1.49 −1.24), p= 0,085), respectively),  The intervention had a statistically significant effect on child development outcomes between the IG and CG at 24 months (cognitive: 70.18 (8.71; 69.18–71.18); language: 18.25 (4.86; 17.70–18.81); motor: 53.36 (5.21; 52.76–53.96), p < 0.05).  The effect of the intervention was maximum for mother-child interaction at 24 months (0.4, 95% CI, 0.22–0.58, p<0.001). | Adequate | Adequate |
| Hossain et al. | 2024 | Bangladesh | CRCT | Intervention | Good health  Adequate nutrition  Opportunities for early learning  Responsive caregiving  Safety and security | Mothers who had a child aged 6–16 months | Child development  -Cognitive, language and motor development-  (Bayley Scales of Infant and Toddler Development third edition)  Children's growth  Food Insecurity  Malnutrition | After one year, the intervened children had higher cognitive [Effect size Cohen's d; 0.42 SD (95% CI: 0.58–0.25)], language (0.38 SD, 95% CI: 0.55–0.22) and motor (0.17 SD, 95% CI: 0.01–0.34) development.  In the intervention group, mothers experienced less violence [OR; 0.6 (95% CI: 0.4–1.0)], and fathers engaged more (0.23 SD, CI: 0.39–0.06) in ECD activities with their children compared to the comparison group.  Total home stimulation and mothers' childcare knowledge were also improved in the intervention.  The children's growth was not improved. | Adequate | Adequate |
| Koshy et al. | 2024 | India | Prospective cohort | Intervention | Opportunities for early learning | Newborns to 9 years old | Child development  -Cognitive development-  (Weschler’s Preschool Primary Scale of Intelligence (WPPSI) and Malin’s intelligence scale for Indian children (MISIC)) | At 5 years, structured early childhood education (ECE) of 18 to 24 months duration was significantly associated with higher cognition scores, with the highest increase in processing speed [β: 19.55 (11.26–27.77)], followed by full-scale intelligence [β: 6.75 (2.96–10.550)].  Bivariate analysis of cognition scores across tertile categories of structured ECE showed that children in the highest tertile (18–24 months of attendance) had significantly higher IQ scores than those in the lowest tertile (null attendance to structured preschool).  Children exposed to 13–24 months of ECE significantly increased full-scale IQ by 4.8 compared to those who did not attend structured ECE [β: 4.82 (2.1–7.5)]. | Moderate | Low |

*CRCT: Cluster randomized controlled trial; RCT: Randomized controlled trial; ASQ: Ages & Stages Questionnaire; LAZ: length-for-age Z score; WAZ: weight-for-age Z-score; HAZ: height-for-age Z score; WHZ: Weight-for-height Z-score; MUAC: Mid-upper arm circumference. ^1^Qualitative study.
